# Evaluation of the psychometric properties of self-reported measures of alcohol consumption: a COSMIN systematic review

**DOI:** 10.1186/s13011-018-0143-8

**Published:** 2018-02-02

**Authors:** Hannah McKenna, Charlene Treanor, Dermot O’Reilly, Michael Donnelly

**Affiliations:** 1 Centre for Public Health, School of Medicine, Dentistry and Biomedical Sciences, Institute of Clinical Sciences – Block B, Royal Victoria Hospital site, Queen’s University Belfast, BT12 6BJ Belfast, Northern Ireland; 2 UKCRC Centre of Excellence for Public Health (Northern Ireland), Queen’s University Belfast, Belfast, Northern Ireland; 3 Administrative Data Research Centre (Northern Ireland), Queen’s University Belfast, Belfast, Northern Ireland

**Keywords:** Self-reporting alcohol intake, Psychometric properties, COSMIN systematic review

## Abstract

**Purpose:**

To review studies about the reliability and validity of self-reported alcohol consumption measures among adults, an area which needs updating to reflect current research.

**Methods:**

Databases (PUBMED (1966-present), MEDLINE (1946-present), EMBASE (1947-present), Cumulative Index of Nursing and Allied Health Literature (CINAHL) (1937-present), PsycINFO (1887-present) and Social Science Citation Index (1976-present)) were searched systematically for studies from inception to 11th August 2017. Pairs of independent reviewers screened study titles, abstracts and full texts with high agreement and a third author resolved disagreements. A comprehensive quality assessment was conducted of the reported psychometric properties of measures of alcohol consumption using the COnsensus-based Standards for the selection of health Measurement Instruments (COSMIN) to derive ratings of poor, fair, good or excellent for each checklist item relating to each psychometric property.

**Results:**

Twenty-eight studies met inclusion criteria and, collectively, they investigated twenty-one short-term recall measures, fourteen quantity-frequency measures and eleven graduated-frequency measures. All measures demonstrated adequate/good test-retest reliability and convergent validity. Quantity-frequency measures demonstrated adequate/good criterion validity; graduated-frequency and short-term recall measures demonstrated adequate/good divergent validity. Quantity-frequency measures and short-term recall measures demonstrated adequate/good hypothesis validity; short-term recall measures demonstrated adequate construct validity. Methodological quality varied within and between studies.

**Conclusions:**

It was difficult to discern conclusively which measure was the most reliable and valid given that no study assessed all psychometric properties and the included studies varied in the psychometric properties that they selected to assess. However, when the results from the range of studies were considered and summed, they tended to indicate that the quantity-frequency measure compared to the other two measures performed best in psychometric terms and, therefore, it is likely to produce the most reliable and valid assessment of alcohol consumption in population surveys.

**Electronic supplementary material:**

The online version of this article (10.1186/s13011-018-0143-8) contains supplementary material, which is available to authorized users.

## Background

Alcohol use and associated consequences are a major public health problem, described as the third leading risk factor for poor health globally [1]. Recently, new revised guidelines from UK (United Kingdom) Chief Medical Officers advised adults about the likely harmful health effects of drinking more than 14 units/week [2], which is approximately six 175 ml glasses of (13%) wine, six 568 ml pints of (4%) lager or ale or (4.5%) cider or fourteen 25 ml measures of (40%) spirts (1 unit is 10 ml or 8 g of pure alcohol) in the UK [3]. The Global Burden of Disease Survey identified alcohol as a top five risk factor for non-communicable disease in the UK [4]. It is important that reliable and valid measures are used to monitor and assess alcohol misuse and related problems and, in turn, to inform public health strategies.

Our initial scoping exercise indicated that data about alcohol intake tends to be collected in surveys using one or more of the following three types of self-report questionnaires: *Quantity-frequency* measures ask questions about ‘usual’ alcohol drinking to estimate the frequency (e.g. number of days per week) and volume of alcohol consumed (e.g. ‘how many (cans/bottles/ glasses) were consumed on a typical drinking day’ [5–7]). *Graduated-frequency* questionnaires measure the volume of consumed alcohol by grouping the number of drinks per occasion into graduated categories, beginning typically with the highest amount consumed by a respondent and decreasing in pre-set categories (e.g. ‘During the last 12-months, how often did you have 12 or more drinks of any kind of alcoholic beverage in a single day?’ ‘During the last 12 months, how often did you have at least 8 but less than 12 drinks of any kind of alcoholic beverage in a single day?’ [8, 9]). *Short-term recall measure*s ask respondents to recall the alcohol that they consumed within a predetermined timeframe such as during the previous week or the last 24-h (e.g. the ‘Yesterday’ method) or using a diary to record all alcohol consumption over a period of time [10, 11].

There is a need to ensure that survey instruments discern accurately alcohol consumption in order to identify the population of drinkers who consume over 14 units of alcohol per week [2], or misuse alcohol. In this review alcohol misuse is defined as *‘drinking excessively – more than the lower-risk limits of alcohol consumption’* [12]*.* Gmel [13] conducted a literature review of self-report measures (the quantity-frequency, graduated-frequency and short-term recall measures) compared to biological tests (i.e. blood alcohol concentration) using studies published in this field since 2004; and Feunekes [14] conducted a systematic review of studies published 1984–1999 on the capacity of the quantity frequency, extended quantity frequency, retrospective diary, prospective diary, and 24-h recall measures, respectively, to classify individuals according to their alcohol intake. These previous reviews are outdated and not in keeping with advances in survey methodology and design concerning alcohol research or with public health guideline changes (such as the reduction in alcohol guidelines in the UK [2]). This paper presents the results of a systematic review of all relevant research evidence regarding the reliability and validity of different types of survey measures of self-reported alcohol consumption in the adult population. Reliability and validity in this review are defined by the COnsensus-based Standards for the selection of health Measurement Instruments (COSMIN) methodology [15]. COSMIN provided an iterative way of assessing the psychometric properties of included measures. The review adds to previous research by providing the first COSMIN-type review of alcohol intake measures as well as providing an updated review of the alcohol consumption measures. This review addressed the following questions:

Are self-reporting measures (the quantity-frequency, graduated-frequency and short term recall measures) reliable and valid in their assessment of alcohol consumption for the general population? If so, which of the self-reporting measures are most reliable and valid? Which measure most accurately identifies levels of alcohol consumption? The use of a reliable and valid measure in alcohol survey research will enhance the rigour and comparability of studies.

## Methods

**Fig. 1:**
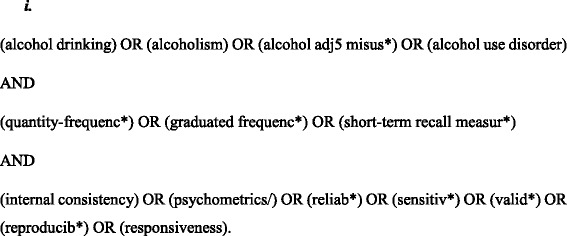
Search strategy; List of free text terms and medical subject headings searched for using the conjunctions ‘AND’ or ‘OR’ to find articles which met the inclusion criteria using the online bibliographic databases

The review was reported in accordance with PRISMA guidelines (see checklist attached as Additional file 1) [16]. No protocol exists for this review. Study authors searched PUBMED (1966-present), MEDLINE (1946-present), EMBASE (1947-present), CINAHL (1937-present), PsycINFO (1887-present) and SSCI (1976-present) from their inception to 11th August 2017 for peer-reviewed articles. Search terms were based on a COSMIN search filter to identify studies of psychometric properties, combined with terms relevant to alcohol intake measures (Fig. 1).

### Eligibility criteria

Papers were included if they were English language peer-reviewed studies that evaluated the reliability or validity of survey measures of alcohol consumption that were ‘self-completed’ by adults aged ≥18 years via telephone, paper, computer or interview. Studies were included if they assessed the reliability or validity of self-report alcohol consumption measures (the quantity-frequency, graduated-frequency or short term recall measures or any variation of these measures). Studies were excluded if they did not focus on reliability or validity, were reviews of the literature or study participants had a mental or alcohol disorder diagnosis, were in receipt of treatment for alcohol misuse or were being cared for in a care institution. The review focused upon evaluating the psychometric properties of alcohol consumption measurement for the general drinking population; previous research indicates that people with an alcohol use disorder diagnosis tend to self-report differently from other drinkers (see discussion [17]). Studies were excluded also if they measured self-reported alcohol consumption using other methods only (biological testing or self-reporting alcohol tests).

**Fig. 2:**
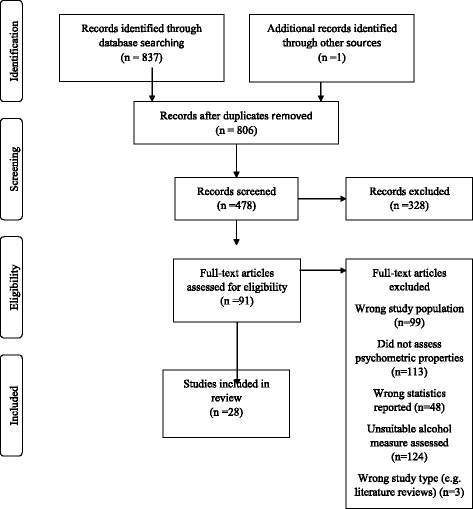
PRISMA flow diagram [16]; Flowchart depicting the process of searching, selecting and sifting studies according to eligibility criteria. The search stages were identification, screening, eligibility and inclusion

Titles were exported to Refworks, duplicates were removed and titles and then suitable abstracts were screened and examined by HMcK, CT and MD independently. Cases of disagreement over study inclusion were resolved via review and discussion. Data collection from eligible studies involved extracting information about population characteristics, measures, results and COSMIN quality ratings onto an Excel spreadsheet (see Table 2). This was completed by HMcK and checked by other reviewers. Reference lists of literature reviews and citation lists of included studies were searched for relevant papers. The search strategy identified 806 studies after duplicate removal, 478 remained following examination of abstracts and 28 papers were included following full-text review (Fig. 2).

### Quality assessment

Pairs of independent reviewers applied the well-validated COSMIN checklist to assess the methodological quality of included studies. Definitions of the psychometric properties are provided by COSMIN (see Table 1). Information (e.g. coefficients) on psychometric properties reported on each measure by included studies were assessed using the quality criteria COSMIN checklist created by Terwee [18] which generated ratings of good, moderate or poor. An additional methodological quality score was calculated for each psychometric property checklist using the ‘worst score counts’ method, where the lowest rating of any of the items in an individual psychometric property checklist is taken as the overall score for that property [19]. Risk of bias (where evidence reported by studies may not be trustworthy [20]) was accounted for by assessing methodological quality of studies. It is important to note that the review reported the properties that were recorded in the original articles and that most articles did not assess or report the full range of properties recommended by COSMIN.

**Table 1:** COSMIN definitions of domains, measurement properties, and aspects of measurement properties [18]

Term			Definition
Domain	Measurement property	Aspect of a measurement property	
Reliability			The degree to which the measurement is free from measurement error
Reliability (extended definition)			The extent to which scores for patients who have not changed are the same for repeated measurement under several conditions: e.g. using different sets of items from the same health related-patient reported outcomes (HR-PRO) (internal consistency); over time (test-retest); by different persons on the same occasion (inter-rater); or by the same persons (i.e. raters or responders) on different occasions (intra-rater)
	Internal consistency		The degree of the interrelatedness among the items
	Reliability		The proportion of the total variance in the measurements which is due to ‘true’^a^ differences between patients
	Measurement error		The systematic and random error of a patient’s score that is not attributed to true changes in the construct to be measured
Validity			The degree to which an HR-PRO instrument measures the construct(s) it purports to measure
	Content validity		The degree to which the content of an HR-PRO instrument is an adequate reflection of the construct to be measured
		Face validity	The degree to which (the items of) an HR-PRO instrument indeed looks as though they are an adequate reflection of the construct to be measured
	Construct validity		The degree to which the scores of an HR-PRO instrument are consistent with hypotheses (for instance with regard to internal relationships, relationships to scores of other instruments, or differences between relevant groups) based on the assumptionthat the HRPRO instrument validly measures the construct to be measured
		Structural validity	The degree to which the scores of an HR-PRO instrument are an adequate reflection of the dimensionality of the construct to be measured
		Hypotheses testing	Idem construct validity
		Cross-cultural validity	The degree to which the performance of the items on a translated or culturally adapted HR-PRO instrument are an adequate reflection of the performance of the items of the original version of the HR-PRO instrument
	Criterion validity		The degree to which the scores of an HR-PRO instrument are an adequate reflection of a ‘gold standard’
Responsiveness			The ability of an HR-PRO instrument to detect change over time in the construct to be measured
	Responsiveness		Idem responsiveness
Interpretability^b^			Interpretability is the degree to which one can assign qualitative meaning - that is, clinical or commonly understood connotations – to an instrument’s quantitative scores or change in scores.

Table Legend: Table of definitions of psychometric properties measured by the COSMIN checklist, grouped by property (e.g. reliability, validity, responsiveness and interpretability)

^a^The word ‘true’ must be seen in the context of the CTT, which states that any observation is composed of two components – a true score and error associated with the observation. ‘True’ is the average score that would be obtained if the scale were given an infinite number of times. It refers only to the consistency of the score, and not to its accuracy [54]

^b^Interpretability is not considered a measurement property, but an important characteristic of a measurement instrument

## Results

Table 2 presents the characteristics and results from the 28 papers that met inclusion criteria. It acts as a summary of the content from Additional file 2: Tables S1 and S2 which are included as Additional files 2 and 3. Included studies reported drinks/alcohol measures in standard sizes for the country of publication (see Additional file 2: Table S1). Some studies included beverage specific measures. Studies were conducted in the USA (*n* = 18), Australia (*n* = 4), Canada (*n* = 2), Finland (n = 2), UK (n = 1) and the Netherlands (n = 1). Most studies included short-term recall measures (*n* = 21), quantity-frequency measures (*n* = 14) and graduated-frequency measures (*n* = 11). Convergent validity (*n* = 15), criterion validity (*n* = 14), test-retest reliability (*n* = 10), predictive validity (*n* = 9), inter-rater reliability (*n* = 5), hypothesis validity (n = 4), construct validity (*n* = 2), divergent validity (n = 2), and structural validity (*n* = 1) were assessed across the studies. Some studies assessed the psychometric properties of more than one measure and measure type but not one study assessed all COSMIN psychometric properties.

**Table 2:** Summary of characteristics and psychometric properties for included studies

Author (country)	Study Population	Methods used	Studies and measures	Psychometric properties reported by studies	COSMIN quality ratings
Bonevski et al. (2010) Australia	Group 1 was 30% male and 70% female, Group 2 37% male and 63% female, Group 3 44% male and 56% female and Group 4 41% male and 59% female. Group 1 mean age 25 years. Group 2 mean age 27 years. Group 3 mean age 25 years. Group 4 mean age 25 years.	Participants were asked to recall alcohol intake using either a computer or paper administered measure. 4–7 days later both modes of measures were administered again.	Weekly quantity-frequency measure.	Test-retest reliability-kappa coefficient range (0.90–0.96). Test-retest reliability was good.	Test-retest reliability (poor)
Chaikelson et al. (1994) Canada	Random sampling was used. The sample was 100% male with mean age 69 years. Wives were also asked same questions via written questionnaire to assess concordance.	Results compared to alcohol test the MAST (Michigan Alcoholism Screening Test [55]) for reliability and validity.	Short-term recall measure (drinking occasions in the previous month recall).	Test-retest reliability- kappa coefficients (0.76) total lifetime drinking, (0.84) last reported month and (0.77) monthly alcohol consumption indicating good test-retest reliability. Concurrent validity- correlations between self-reports (0.87) husband alcohol intake and (0.85) wife alcohol intake indicating good criterion validity. Construct validity- correlations with the MAST self-report test in 1987(0.60) with total lifetime drinking (0.05) with current drinking. Correlations with 1990 data (0.53) with total lifetime drinking (− 0.14) with current drinking. Construct validity shows moderate reported correlation.	Test-retest reliability (fair) Criterion validity (poor) Construct validity (poor)
Crum et al. (2002) USA	Random sampling was used. The sample was 58% female and 42% male with mean age 76.2 years. Data was obtained from the 1993–1994 follow-up of the Washington County cohort of men and women 65 years and older.	Participants completed a measure of their usual alcohol consumption in two ways: (1) a quantity-frequency measure; (2) same questions asked in an interview about drinking habits.	Weekly quantity-frequency measure. Short-term recall measure (past week recall).	Hypothesis validity-past week recall of alcohol intake 15–20% lower than the quantity-frequency measure. Hypothesis validity was good. Inter-rater reliability-kappa statistic value 0.76 indicating good inter-rater reliability.	Hypothesis validity (good) Inter-rater reliability (poor)
Cutler et al. (1988) UK	Random sampling was used. 63.4% of the sample were male and 36.6% female. No median or mean age was reported but participants were aged 18 and older.	CAGE responses and the quantity-frequency questions taken from Health Survey Questionnaire were compared.	Weekly quantity-frequency measure.	Criterion validity-sensitivity (42.9) specificity (97.1) positive predictive value (65.8) negative predictive value (92.8) for males and sensitivity (46.6) specificity (98.6) positive predictive value (50.3) negative predictive value (98.4) for females indicating good criterion validity.	Criterion validity (excellent)
Dollinger et al. (2009) USA	The sample was composed of volunteers and was 61% female and 39% male with a mean age 22 years.	Responses to quantity-frequency measures at both time points compared. Nightly log of alcohol consumption compared to hours spent studying, socialising and religious behaviours.	Daily graduated-frequency measure. Short-term recall measure (daily alcohol intake recall).	Test-retest reliability-alcohol quantity coefficient of 0.85 and an alcohol frequency coefficient of 0.84 indicating good test-retest reliability. Divergent validity-religion-by-alcohol correlations were negative with values from −0.14 to −0.37. Convergent validity-positive correlations with alcohol with values of 0.40 and 0.41 respectively. Good divergent and convergent validity were reported.	Test-retest reliability (fair) Divergent validity (fair) Convergentvalidity (fair)
Greenfield et al. (2014) USA	Random sampling was used. Respondents were 48.1% male and 53.2% female and aged over 18 years.	Participants completed questionnaires and a follow-up survey by phone or mail.	Short-term recall measure (occasions of ≥5 drinks during specific life decades).	Test-retest reliability-kappa values for gender (0.64–0.80), age groups (0.59–0.83), ethnicity (0.70–0.73), interview mode (0.72–0.73) and childhood victimisation (0.75) (0.73) indicating moderate to good test-retest reliability. Predictive validity-disclosure of prior heavy drinking increased risk for alcohol dependence by 18%, increased risk of consequences by 21% (by 15% when age of onset was controlled), increased risk for alcohol-use disorder by 18% indicating good predictive validity.	Test-retest reliability (fair) Predictive validity (fair)
Gruenewald et al. (1995) USA	Random sampling was used. Respondents were 43.5% male and 56.5% female and aged 18 years or older.	Responses to graduated-frequency measures at two time points compared.	Gruenewald et al. (1995) Monthly graduated-frequency measure	Test-retest reliability-coefficients for average drinking quantity *r* = 0.76 and for variance in drinking quantities *r* = 0.78, indicating good test-retest reliability.	Test-retest reliability (fair)
Hansell et al. (2008) Australia	Random sampling was used. Respondents were 40% male and 60% female and aged between 19 and 90 years old.	The measures examined were a dependence score, based on DSM-IIIR (Diagnostic and Statistical Manual of Mental Disorders [56]) and DSM-IV criteria for substance dependence, and a quantity × frequency of alcohol consumed taken from the quantity-frequency measure.	Annual quantity-frequency measure	Test-retest reliability-continuous data quantity x frequency of alcohol (0.61) between phase 1 and phase 3, and (0.55) between phase 2 and phase 3. Categorical data quantity x frequency of alcohol (0.64) between phase 1 and phase 3, and (0.59) between phase 2 and phase 3, indicating moderate test-retest reliability.	Test-retest reliability (poor)
Hilton (1989) USA	Volunteer sample. Respondents were 50% male and 50% female and had a mean age of 30 years. The volunteer participants were recruited from the San Francisco Bay Area newspaper.	Participants completed 2 retrospective recall measures-graduated-frequency and beverage-specific quantity-frequency measures post diary completion. Responses compared.	Short-term recall measure (10 week recall). Graduated-frequency measure (30 day recall). Beverage specific Quantity-frequency measure (2 week recall).	Convergentvalidity-correlations 0.88 for volume of drinks consumed, 0.85 for days of beer consumed, 0.89 for days of beer usually consumed, 0.80 for days of wine consumed, 0.66 for days of wine usually consumed, 0.81 for days of liquor consumed and 0.65 for days of liquor usually consumed, indicating moderate to good convergent validity.	Convergent validity (fair)
Koppes et al. (2002) Netherlands	Random sampling was used. Respondents were 46% male and 54% female with mean age 36 years. Data was collected from 1 time point, the 2000 follow-up measurement of 171 male and 197 female participants from the Amsterdam Growth and Health Longitudinal Study.	Subjects visited study premises for 1 day. The quantity-frequency measure and dietary history interview were based on alcohol consumption over the previous month and were completed in no particular order.	Quantity-frequency measure (ranging from never drinking to daily alcohol intake). Short-term recall measure (dietary history interview).	Concurrent validity-correlation between (0.77) for men and (0.87) for women, which indicates good concurrent validity.	Criterion validity (poor)
LaBrie et al. (2004) USA	The sample was composed of volunteers and was 100% male with a mean age of 20.6 years. 211 male college students participated.	Drinking variables assessed were drinking days, average drinks, and total drinks during a 30-day period.	Short-term recall measure (monthly TimeLine follow back method).	Convergentvalidity-correlation coefficients between 0.52–0.69 showing moderate convergent validity.	Convergent validity (fair)
Lennox et al. (1996) USA	Analysis was conducted of a sample of a household survey aged 18–64 years. Gender proportions were not reported. Responses were analysed from 1 time point (the 1991 follow-up) from 8755 participants in the 1988 National Household Survey of Drug Abuse.	Used a latent variable approach. In this model covariation among multiple indicators was used as an estimate of the latent construct.	Quantity-frequency measure of alcohol consumption over past 30 days.	Structural validity-correlations at 0.36, alcohol abuse and consequences between constructs correlates at 0.28 showing poor structural validity.	Structural validity (fair)
McGinley et al. (2014) USA	A sample of 18–20 year olds were selected from respondents to the National Survey on Drug Use and Health. Gender proportions were not reported.	Quantity and frequency of alcohol consumption estimates derived from graduated-frequency measure. Estimates compared to the quantity-frequency measure.	Graduated-frequency measure of alcohol consumption over past 30 days.	Construct validity-mid values for quantity of alcohol consumed were (3.5) and (14.5) for frequency indicating poor construct validity.	Construct validity (fair)
Northcote and Livingston (2011) Australia	Respondents were 47.3% male and 53.3% female and aged 18–25 years.	Participants reported number of alcoholic drinks consumed 1–2 days after drinking occasion which was compared to reported alcohol intake observed by peer-based researchers on the occasion.	Short-term recall measure (last occasion self-report of drinks consumed).	Criterion validity-significant associations with *p* values of 0.6, 0.31, 0.04 and < 0.01 for: up to 4 drinks, 5–8 drinks, 9–12 drinks and more than 12 drinks respectively indicating good criterion validity for respondents consuming ≥9 drinks. . Convergent validity- significant at 0.74, with gender specific correlations formen as 0.79 and women 0.60. Moderate to good convergent validity was reported.	Criterion validity (poor)
O’Hare et al. (1991) USA	Respondents were 41.6% female 58.4% male and with mean age 20.6 years.	Participants were asked to complete mailed questionnaire with both measures of alcohol consumption included.	Weekly graduated-frequency measure. Short-term recall measure (retrospective recall of past 7 day alcohol intake).	Convergent validity-correlations were significant at 0.74, with gender specific correlations for men as 0.79 and women 0.60, indicating moderate to good convergent validity.	Convergent validity (good)
O’Hare et al. (1997) USA	Random sample of an undergraduate university population. Gender proportions were reported as ‘representative of sex’. Respondents had a mean age of 18.7 years.	All students completed quantity-frequency questions, MmMAST and 7 day recall. The MmMAST was used as a criterion variable.	Weekly graduated-frequency measure. Short-term recall measure (retrospective recall of past 7 day alcohol intake).	Criterion validity-association was significant at *p* < 0.01 indicating good criterion validity. Predictive validity-sensitivity and specificity values were 76 and 59.8 for the recall measure. Using MAST cut off score ≥ 2 sensitivity and specificity values were 59.7 and 70.9 indicating moderate to good predictive validity.	Criterion validity (fair) Predictive validity (fair)
Parker et al. (1996) USA	Random sampling was used. Respondents were 39% male and61% female and aged 18–64. Data was taken from surveys 1987–1989, 1989–1990 and 1992–1993 of the Pawtucket Health Program conducted among home dwelling adults.	Alcohol intake assessed with food frequency question as a component of the general health survey was compared against alcohol intake assessed with a graduated-frequency measure as part of a survey.	Short-term recall measure (beverage specific past 24 h recall). Annual graduated-frequency measure	Concurrent validity-kappa statistics reported between measures ranged from 0.08 (*p* < 0.001), 0.38 (*p* < 0.001) and 0.81 (*p* < 0.001), indicating good concurrent validity for high consumers of alcohol only. Inter-rater reliability Kappa values for both measures were (0.28–0.47). Inter-rater reliability was poor (below 0.70).	Criterion validity (poor) Inter-rater Reliability (fair)
Poikolainen et al. (2002) Finland	Volunteer sample recruited from their workplace. Respondents were 83% female and 17% male with a mean age of 42 years.	Quantity-frequency and graduated-frequency obtained before and after 1-month daily recall on alcohol intake. Blood sample obtained at outset.	Annual quantity-frequency questionnaire. Daily graduated-frequency measure. Short-term recall measure (past month recall of intake).	Convergent validity-coefficients were 0.95 between the short-term recall measure and quantity-frequency 1, 0.95 between the short-term recall measure and quantity-frequency 2, 0.90 between the short-term recall measure and graduated-frequency 1 and 0.93 between the short-term recall measure andgraduated-frequency 2. Convergent validity was reported as good.	Convergent validity (good)
Read et al. (2006) USA	College students who reported drinking different amounts of alcohol were selected for the sample to be representative of variation in drinking levels. Respondents were 52% female and 48% male with a mean age 19 years.	College students completed self-report questionnaire on demographic characteristics, drinking behaviours and drinking consequences. Drinking consequences assessed with composite measure based on Drinker Inventory of Consequences and Young Adult Alcohol Problem Screening Test developed by researchers.	Short-term recall measure (past 90 day intake).	Concurrent validity-correlation values of 0.36, *p* < 0.001 and with quantities of alcohol consumed with anr value of 0.31, *p* < 0.001, indicating poor concurrent validity.	Criterion validity (excellent)
Rehm et al. (1999) Canada	The sample was chosen to be representative of the wider drinking population. Respondents were 48% male and 52% female, and chosen to be representative of age ≥ 18 years.	Population samples from 4 surveys conducted for Alcohol Research Group. Surveys used computer-assisted telephone interviews with random digit dialling sampling techniques.	Quantity-frequency measure for drinking occasion. Annual Graduated-frequency measure. Short-term recall measure (past week recall.	Convergent validity-correlations moderate at both approximately 0.40. Predictive validity-estimates by graduated-frequency measure 22% higher than short-term recall estimate. Quantity-frequency estimate of alcohol-related mortality 13% than short-term recall estimate, indicating poor predictive validity.	Convergent validity (fair) Predictive validity (excellent)
Reid et al. (2003) USA	Random sampling was used. The veteran primary care sample was 3% female 97% male and the community dwelling sample was 60% female 40% male. Mean ages were 73.1 for the veteran primary care sample and 75.9 for the community dwelling sample.	Telephone call allowed self-report of quantity-frequency measure, binge and heavy drinking questions, and the AUDIT (Alcohol Use Disorders Identification Test [44]) and CAGE (Cut down, Annoyed, Guilty, Eye-opener [45]) tests.	Weekly quantity-frequency measure.	Inter-rater reliability-kappa values were 0.44 and 0.33. For population sample 2 kappa values were 0.21 and 0.46 indicating moderate to poor inter-rater reliability.	Inter-rater Reliability (fair)
Russell et al. (1991) USA	Random sampling was used. Respondents were 50.5% male and 49.5% female and aged over 18 years. Data was taken from 1 time point of the survey.	Quantity-frequency questions were asked about the amount and frequency of particular alcoholic beverages consumed via telephone interview using a random-digit-dial technique and supplemented by samples of homeless people, college students and those without telephones.	Typical annual beverage-specific Quantity-frequency measure	Criterion validity-correlations between 0.73 and 0.77 for subtypes of alcohol reported showing good criterion validity.	Criterion validity (poor)
Sander et al. (1997) USA	175 patients with traumatic brain injury were recruited from a medical rehabilitation centre along with their relatives. Respondents were 65% male and 35% female. Mean age 39.2 years for patients and 45.9 years for relatives.	Alcohol use examined 1 year after injury through quantity-frequency measure and brief MAST test. Patients and their relatives both completed measures and concordance between reports were examined.	Annual quantity-frequency measure	Concurrent validity-concordance showed 95.4% agreement indicating good criterion validity.	Criterion validity (fair)
Searles et al. (1995) USA	The sample was chosen to be representative of male drinking population in Vermont enrolled in the Alcohol Research Centre. Respondents had a median age of 28 years(ranging from 21 to 56 years) and were 100% male.	Subjects self-reported daily alcohol intake via telephone.At 90days subjects completed an interview using DSM criteria to assess alcohol abuse ordependence.	Short-term recall measure (Daily self-report of alcohol intake). Short-term recall measure (annual retrospective recall).	Predictive validity-correlations0.86 andwith alcohol related problems level as 0.69. Predictive validity is moderate between daily self-report and retrospective recall and alcohol related problems, and good between daily self-report and retrospective recall and alcohol intoxication level.	Predictive validity (poor)
Searles et al. (2000) USA	Volunteer sample of those enrolled in the Vermont Alcohol Research Centre. Respondents were 100% male and had a mean age of 36.2 years for those without alcohol problems tested at outset and 30.4 years for those with alcohol problems.	Participants recorded alcohol intake on interactive voice response system using telephones. In person interviews were conducted every 13 weeks during which they completed timeline follow back. Results were compared.	Short-term recall measure (Timeline Follow back over 366 days). Short-term recall measure (Daily self-report of alcohol intake).	Convergent validity-correlations 0.60 at 180 days of administration, 0.57 at 270 days of administration and 0.57 at 366 days of administration, indicating moderate convergent validity.	Convergent validity (fair)
Tuunanen et al. (2013) Finland	The sample included 45 year olds resident in Finnish city of Tampere. The sample was 100% male.	Participants completed a mailed health questionnaire which invited previous week recall of alcohol intake, a quantity-frequency measure and structured quantity-frequency questions based on the AUDIT.	Quantity-frequency measure (typical drinks consumed per occasion). Short-term recall measure (past week recall).	Hypothesis validity-the past week recall measure reported mean alcohol consumption lower than the quantity-frequency measure indicating good hypothesis validity.	Hypothesis validity (fair)
Weingardt et al. (1998) USA	Random sampling was used. Respondents were 58% female and 42% male and aged 18–20 years.Data was taken from 1990 and 1994 cohorts of college undergraduate students.	Peak consumption, typical weekend quantity and typical daily quantity measures used to derive binge drinking data to analyse validity. Binge drinking defined as 5–6 drinks per occasion for men and 3–4 drinks per occasion for women.	Graduated-frequency measure (peak monthly alcohol consumption). Graduated-frequency measure (typical weekend quantity). Short-term recall measure (typical daily quantity).	Concurrent validity-r value 0.57 and Alcohol Dependence Scale with r value 0.54. Predictive validity-daily quantity measure classified 6.2% of drinkers as chronic and 7.4% indicating poor predictive validity.	Criterion validity (good) Predictive validity (good)
Whitfield et al. (2004) Australia	Voluntary sample. Respondents were 36% male and 64% female with a mean age of 33.7 years. Data was taken from 3 waves (1980, 1989 and 1993) using adult male and female participants of the AustralianTwin Registry.	Test-retest reliability was calculated as correlations between occasions and between measures. Relationships between alcohol use and lifetime DSMIIIR alcohol dependence examined.	Annual quantity-frequency measure. Short-term recall measure (past week recall of alcohol intake).	Test-retest reliability-correlations between (0.54–0.70) indicating moderate to good test-retest reliability.	Test-retest reliability (fair)

Table Legend: Table summarising the characteristics, findings and COSMIN quality ratings of included studies grouped by study author, study population, methods used, studies and measures, psychometric properties reported by study authors and COSMIN quality ratings

### Methodological quality assessment

There was wide variation in methodological quality ratings for each psychometric property (as presented and discussed below).

Quantity-frequency measures achieved criterion validity ratings of excellent (*n* = 1), fair (n = 1) and poor (*n* = 2). Test-retest reliability quality ratings were good (*n* = 1), fair (*n* = 1) and poor (n = 2), with inter-rater reliability rated fair (*n* = 1) and poor (*n* = 1). Convergent validity ratings were good (*n* = 1) and fair (n = 2). Hypothesis validity was rated good (n = 1) and fair (*n* = 1). Predictive validity was rated excellent (*n* = 1) and structural validity fair (n = 1).

The graduated-frequency measures achieved convergent validity ratings of good (*n* = 2) and fair (*n* = 3). Test-retest reliability ratings were rated fair (*n* = 2) and good (*n* = 1) and inter-rater reliability was also rated fair (*n* = 1). Criterion validity was rated good (*n* = 1), fair (n = 1) and poor (n = 1). Predictive validity was rated excellent (*n* = 1), good (*n* = 1) and fair (n = 1). Divergent validity was rated fair (n = 1). Construct validity was rated fair (n = 1).

The criterion validity ratings for the short-term recall measures were excellent (n = 1), good (n = 1), fair (n = 1) and poor (*n* = 4). Convergent validity was rated good (n = 2) and fair (*n* = 5). Predictive validity was rated excellent (n = 1), good (n = 1), fair (n = 2) and poor (n = 1). Test-retest reliability scores were rated fair (*n* = 3), with inter-rater reliability also rated fair (n = 1). Hypothesis validity was rated good (n = 1) and fair (n = 1). Divergent validity was rated fair (n = 1) and construct validity was rated poor (n = 1).

### Test-retest reliability

Quantity-frequency and graduated-frequency measures completed by a Finnish population sample [11] and a computer and paper administered quantity-frequency measure demonstrated good test-retest reliabilities [6]. Moderate test-retest reliabilities were reported for a quantity-frequency measure administered to a general population sample [21] and for quantity-frequency and short-term recall measures in an Australian general sample of twins [22]. Good test-retest reliability was reported in an undergraduate student population sample for a graduated-frequency measure [10] and in a general population [23]. Test-retest reliability of a daily intake short-term recall measure was good for an older adult sample [24]. Moderate test-retest reliability was reported for a short-term recall measure of ≥5 drinks consumed per drinking occasion [25]. In an older population sample, inter-rater reliability was good for quantity-frequency and short-term recall measures [26] though poor inter-rater reliability was reported in a study administering a weekly quantity-frequency measure to over 65-year olds [7] and for the graduated-frequency and short-term recall measures in a general population [27] (for detailed results see Table 2).

### Criterion validity

Studies of quantity-frequency measures administered to the general population sample [28–30] and a quantity-frequency and short-term recall measure [31] demonstrated good criterion validity. An annual graduated-frequency measure and previous 24 h short-term recall measure administered in a general population sample indicated good criterion validity for ‘heavy drinkers’. Poor validity was reported for moderate drinkers in this study (due perhaps to the fact that consumers of lower levels of alcohol may drink irregularly and not within the 24-h before administration of the short-term recall measure) [27]. An undergraduate student sample completed two graduated-frequency measures and a short-term recall measure with moderate criterion validity [32]. Short-term recall spousal reports that were used as a criterion or standard to validate alcohol intake in an older sample reported good criterion validity [24]. A short-term recall measure administered to an undergraduate student sample had poor criterion validity [33] though other studies of the short-term recall measure [34] and the short-term recall and graduated-frequency measures [9] reported good criterion validity **(**see Table 2**)**.

### Construct validity

Poor construct validity was found for 30-day graduated-frequency measure completed in an undergraduate sample (age range 18–20 years) [35]. A short-term recall measure compared with the MAST measure on two separate occasions in a sample of older adults reported poor to moderate construct validity [24] (see Table 2).

### Hypothesis validity

Good hypothesis validity was reported for a quantity-frequency measure compared to a short-term recall measure in an older adult population sample [26] and for a quantity-frequency measure compared to a short-term measure in a general population sample [36] (see Table 2).

### Predictive validity

One study of a graduated-frequency and short-term recall measure that was completed by an undergraduate student sample demonstrated adequate to good predictive validity [9] whilst another (albeit small sample size) study of the same measures in an undergraduate student sample (age range 18–20 years) recorded poor predictive validity [32]. A general population study found poor predictive validity for the three measures [37] though measured against unstandardized indicators of alcohol-related mortality, morbidity and harm. A short-term recall measure achieved good or adequate prediction properties regarding heavy drinking (≥5 drinks per occasion) for samples aged 18–39 [25] and for a general population [38] (see Table 2).

### Convergent validity

Moderate to good convergent validity was found in a general population sample for a two-week beverage-specific quantity-frequency measure, a graduated-frequency and short-term recall measure [39]. Similarly, adequate or good convergent validity was recorded for the three types of measures of alcohol intake in a cohort of 20 to 63-year olds [11] and in a general population [37]. A graduated-frequency and short-term recall measure demonstrated good convergent validity in an undergraduate student samples [8, 10]. A short-term recall measure completed by undergraduate student samples reported adequate to good convergent validity [40]. Also, adequate convergent validity was found for short-term recall measures in a male population sample [41] (see Table 2). Only one study referred to divergent validity of the graduated-frequency and short-term recall measures and only in terms of a negative correlation in an undergraduate student sample between religiosity and alcohol consumption [10] (see Table 2). Similarly, only one study referred explicitly to structural validity - a 30-day quantity-frequency measure that was used to collect data on alcohol consumption in a general population reported poor validity [42] (see Table 2).

Overall, the review found that only a relatively small number of studies investigated the COSMIN psychometric domains of each type of measure. Furthermore, the hypothesis validity or structural validity of the graduated-frequency measure was not investigated at all nor was the structural validity of the short-term recall measure. Divergent validity or construct validity were not assessed for the quantity-frequency measure.

## Discussion

### Psychometric property ratings for measure types

Each type of measure appeared to have good criterion validity according to COSMIN methodology. Several different reference standards or criterions were used in the included studies to measure alcohol consumption (e.g. [9, 29]). The appropriateness of using peers [34], spousal reports [24] and short-term recall measures [31] as criterion standards is questionable and perhaps it is unsurprising that these studies reported a low quality rating (despite reporting good content validity). Currently, there is no gold standard for the measurement of alcohol consumption. Most countries use some standard unit of measurement (e.g. one drink, one unit) but there is a lack of consensus and no internationally accepted definition thereby posing difficulties for the conduct of comparative analyses. Biological markers of alcohol consumption should be used more frequently to support and validate findings from self-reporting measures, as these methods are not subject to sampling errors or researcher or participant bias [14]. However these measures are also not without risk of error. Alcohol abstinence in the 24 h prior to breath-, blood- or urine- ethanol measurement has been shown to produce low results even for heavy drinkers [43]. More research is needed to find a gold standard for alcohol consumption measurement.

Construct validity was poor for graduated-frequency and short-term recall measures, and not assessed for quantity-frequency measures. The structural validity of the quantity-frequency measure only was assessed and this construct validity-related property was deemed to be poor. Only one study investigated the predictive validity of the quantity-frequency measure and it found that the validity was poor. Poor predictive validity results suggest the measure may not be valid in predicting the measurement of future alcohol intake among the general population or in predicting the measurement of drinking trajectories and alcohol-related consequences. The study was conducted with good methodological quality and received a good COSMIN score.

In contrast, the graduated-frequency and short-term recall measures achieved mixed results including predicting with variable accuracy the outcomes of alcohol-related morbidity and mortality and alcohol dependence. There were several studies of the convergent validity of each measure and generally this property was deemed to be moderate to good.

Test-retest results tended to indicate that similar outcome-assessments of alcohol consumption were found when the quantity-frequency measure, graduated-frequency measure and the short-term recall measure were re-administered. Mixed results were reported for inter-rater reliability of quantity-frequency and short-term recall measures, with poor inter-rater reliability found when the graduated-frequency measure was applied. In particular, there appeared to be difficulty obtaining good agreement between raters regarding the measurement of consumed beer, wine and liquor respectively [27], between self-report tests (AUDIT (Alcohol Use Disorders Identification Test [44]) and CAGE (Cut down, Annoyed, Guilty, Eye-opener) [45]) and a quantity-frequency measure when research assistants interviewed participants using a face-to-face predetermined appointment schedule [7]. It is important to note that these studies achieved only fair or poor COSMIN ratings. Indeed, many of the reported poor psychometric properties may be due to poorly conducted studies as indicated by poor COSMIN ratings [6, 21, 31]. Variation between types of psychometric properties for the same measure (e.g. high validity for one property and low for another property) may be due to differences in study design and methodological quality.

### Discrepancies between COSMIN ratings and psychometric properties

There were some studies in which there were discrepancies between COSMIN ratings of the quality of a psychometric property and the performance of a measure. For example, one study [6] reported good test-retest reliability for a typical weekly quantity-frequency measure but the methodological quality of a particular aspect of the study was rated poor because the method of administering the (computer or paper) measure of consumption was not consistent across time-points. Reasons for poor methodological quality ratings using the COSMIN checklist included inappropriate time intervals between measure administrations, ambiguity over management of missing responses, lack of assurance that patients remained stable between measure administrations, inadequate sample size and choice of inappropriate statistical methods (e.g. reporting Spearman’s correlation coefficients [46] over kappa values for test-retest reliability).

### Issues with self-reporting alcohol consumption

Self-reported alcohol consumption is difficult to measure accurately due to the influence of social desirability and memory issues and these factors were alluded to in many included studies (e.g. [25, 27, 32, 35]). Possible solutions to these challenges include using more anonymised interview types, randomised response techniques, checking responses using more than one alcohol measure and using memory aids (interviewer prompts, calendars or diaries) [47]. Also, population-based survey research about alcohol consumption and drinking habits are particularly problematic when the sample includes alcoholics because of uncertainty about whether or not participants are sober when interviewed, difficulty recalling consumption due to the effect of alcohol on memory and increased alcohol tolerance in frequently heavy drinkers [48]. These issues pose challenges for the reliable and valid assessment of alcohol consumption in surveys. Potential solutions include factoring in more complex survey questions requiring greater reflection on alcohol intake (if respondents are asked to consider the timing, type of beverage drank and episodic heavy drinking their responses should be more considered), [17] use of a breathalyser before measure administration to ensure participants are alcohol-free [49] and creating an environment that is conducive to confidentiality and honest disclosure of alcohol consumption [48, 50]. These potential solutions may be incorporated into population-based survey collection of alcohol consumption data in order to afford greater confidence in the drinking status of participants and significant assurance that responses reflect consumption accurately.

### Comparison with previous reviews

Generally, the measures did not appear to vary significantly across population age and sex groupings. The assessment of the amount of alcohol consumed appeared to exert some influence on the psychometric performance of self-report measures. Parker [27] reported good concurrent validity using a short-term recall measure though for heavy drinkers only. Gmel [13] found the graduated-frequency measure over reported alcohol intake, whereas the beverage specific quantity-frequency measure provided a more accurate measure of consumption. The Feunekes review recommended that the quantity and frequency of alcohol consumption should be prioritised and assessed separately for specific types of alcoholic beverages [14] and beverage-specific quantity-frequency measures performed accurately and reliably though only in relation to the consumption of lower levels of alcohol [26, 28]. The use of a ‘diary’ format with a predetermined timeframe (that afforded individuals an opportunity to record all alcohol consumption in a format of their choice; and usually in the format of a short-term recall measure) had good psychometric properties [24, 29]. This finding may suggest that the use of an ‘actual’ time period instead of the ‘usual’ timeframes in quantity-frequency and graduated-frequency measures [51] may add to the reliability and validity of assessments of alcohol consumption. However both reviews found that the quantity-frequency measure performed with most reliability and validity and was the measure with the highest concordance with the short-term recall ‘diary’ measure [22, 29, 33, 38].

### Recommendations for improved reliability and validity

The review findings suggest that the reliability and validity of self-reporting alcohol consumption measures may be improved in various ways. For example, computerised or automated modes of administration rather than an interviewer-based mode might facilitate greater privacy and assure more candid reporting [52]. Longer timeframes may be more desirable as they tend to capture less frequent drinkers (i.e. weekly, monthly or annual recall) and questions which involve specified timeframes (i.e. last week, last year) over ‘usual’ reference frames require respondents to focus their recall. Beverage-specific questions and questions that ask respondents to group responses into graduated categories may encourage a more thorough consideration of their alcohol consumption and, in turn, produce more accurate reporting. It is worth considering that the self-report measures themselves are outdated as they focus only upon frequency and volume of alcohol. It may be worthwhile to instead use self-report tests to assess alcohol consumption which take into account symptoms of alcohol addiction/dependence as well. Using review findings, the advantages and disadvantages of each measure type are summarised (Table 3).

**Table 3:** Summary table of the advantages and disadvantages of the quantity-frequency, graduated-frequency and short-term recall measures

Measure type	Advantages	Disadvantages
Quantity-frequency measures	• Easily administered. • Simple structure; respondents are more likely to understand the measure. • Well-established (respondents are more likely to be familiar with the measure). • Captures ‘usual’ drinking behaviour, unaffected by occasions or seasons where more alcohol consumption may occur. • Can increase reliability by including beverage-specific questions.	• May not record heavy episodic drinking occasions.
Graduated-frequency measures	• Categories act as prompts for respondents. • Answers are easily standardised to identify those drinking above the guidelines. • Can increase reliability by including beverage-specific questions.	• May not record heavy episodic drinking occasions.
Short-term recall measures	• Can focus questions on specific drinking events. • Requires respondents to consider their responses to a greater extent (as answers are not structured). • Respondents can report their alcohol consumption (in standard drinks sizes, units etc.) in a way they are familiar with. • Can increase reliability by including beverage-specific questions.	• Hard to standardise answers to the same measure recorded in different formats. • Respondents may be confused by lack of response options.

Table Legend: Summary of the advantages and disadvantages of the three self-reported alcohol consumption measure types; the quantity-frequency, graduated-frequency and short-term recall measures

### Limitations and strengths

The review found wide variation in the structure, content and format of quantity-frequency, graduated-frequency and short-term recall measures. For example, time-period referents ranged from 24-h recall to alcohol intake over the previous year and alcohol consumption was assessed in terms of units (standardised to the country of each sample of respondents), grams of alcohol, typical sizes of sold drinks and beverage-specific drinks. The included studies from various multidisciplinary databases covered a range of locations, cultures and populations and these factors were taken into account in the analytical comparisons of measures of alcohol consumption. It is important to note that a proportion of the review studies focused on undergraduate student populations (e.g. [8, 10, 34, 40]). Arguably, students may be atypical with respect to the general population [53] and their alcohol consumption patterns may have limited read-across to the general population particularly the population of older people. Some psychometric properties were not assessed including measurement error, cross-cultural validity, internal consistency and responsiveness. All studies were in the English language (in keeping with COSMIN manual guidelines) and it is possible that important studies in other languages may have been missed. The review adhered to the COSMIN manual [15] and whilst the COSMIN method adds rigour to the exercise of psychometric assessment, arguably, a limitation is the use of the ‘worst score counts’ which means that despite attaining higher quality scores on some items, the lowest score of an item list is taken as the overall quality rating (e.g. [28, 31]). Furthermore, studies of poor design quality were included in the review due to the overall lack of studies that met initial eligibility criteria.

Nevertheless, the review was completed in a methodologically robust fashion as per the COSMIN approach which has transparent, tested and validated resources such as a manual, search filters and a quality appraisal tool [15]. Particular strengths include the use of extensive search terms and having two reviewers search the literature.

## Conclusion

The studies of quantity-frequency measures indicated good/adequate psychometric properties for test-retest reliability, criterion validity, convergent validity and hypothesis validity; predictive- and structural-validity were rated as poor and inter-rater reliability reported mixed results. Regarding graduated-frequency measures, good/adequate psychometric properties were reported for test-retest reliability, convergent validity and divergent validity; criterion validity and predictive validity reported mixed results and construct validity and inter-rater reliability were reported as poor. Short-term recall measures achieved good/adequate psychometric properties for test-retest reliability, convergent validity, hypothesis validity, construct validity, divergent validity. Criterion validity, predictive validity and inter-rater reliability reported mixed results. The review findings add to previously published alcohol self-report literature by providing an updated appraisal of measures of alcohol consumption research and indicate that a combination of aspects of the various measures may enhance the reliable and valid assessment patterns of drinking.

It is difficult to discern which one of the existing measures is the most reliable and valid given the absence of any assessment of certain psychometric properties and the mixed results of studies included in the review. Arguably, when the results from the range of studies are considered and summed, they indicate that the quantity-frequency measure compared to the other two measures appeared to perform best in psychometric terms and, therefore, it is likely to produce the most reliable and valid assessment of alcohol consumption in population surveys. The results indicated that the features of alcohol consumption measures which performed with good reliability and validity were those that assessed beverage-specific alcohol consumption, used actual timeframes and asked about episodes of binge drinking; and that the quantity-frequency measures appeared to be the ‘best’ questionnaire-type currently available to measure self-reported alcohol consumption. Clearly, there is a need for more focused psychometric studies of measures of alcohol consumption including head-to-head comparative population-based and community surveys. Comparability of review results with previous reviews [13, 14] is difficult because they did not employ a COSMIN methodology to appraise studies. Overall, findings appeared to be in keeping with the results of the Gmel review [13] which found a beverage-specific, quantity-frequency measure recorded alcohol consumption more reliably, and with the Feunekes [14] which reported that the most accurate alcohol intake measurement was provided by quantity-frequency and short-term recall measures.

## Additional files

Additional file 1: Preferred Reporting Items for Systematic Reviews and Meta-Analyses: The PRISMA statement checklist [16]. Checklist for the minimum required items to be reported as part of a systematic review. (DOC 62 kb)

Additional file 2: Table S1. Characteristics of included studies. A full description of the characteristics of each study which met the review inclusion criteria (*n* = 28). (DOCX 25 kb)

Additional file 3: Table S2. Psychometric properties of included studies grouped into results reported by study authors and COSMIN quality ratings assigned by review authors (n = 28). (DOCX 41 kb)

## Abbreviations

AUDIT: Alcohol use disorders identification test [44]
CAGE: Cut down, Annoyed, guilty, eye-opener (test for problem alcohol use) [45]
COSMIN: Consensus-based Standards for the selection of health measurement instruments [15]
DSM: Diagnostic and statistical manual of mental disorders [56]
MAST: Michigan alcoholism screening Test [55]
DSMIIIR: Diagnostic and statistical manual of mental disorders revised 3rd edition
DSMIV: Diagnostic and statistical manual of mental disorders 4th edition
GF: Graduated-frequency
UK: United Kingdom
