# Supplementary material for: Evaluation of the psychometric properties of self-reported measures of alcohol consumption: a COSMIN systematic review
Source: Subst Abuse Treat Prev Policy. 2018 Feb 2;13:6. doi: 10.1186/s13011-018-0143-8 (PMC5797334; doi:10.1186/s13011-018-0143-8)
Supplement: Supplementary file 2 — Characteristics of included studies. A full description of the characteristics of each study which met the review inclusion criteria (n = 28). (DOCX 25 kb) [file 13011_2018_143_MOESM2_ESM.docx]

Additional file 2 Table S1 Characteristics of studies that met inclusion criteria

| Author (country) | Aim(s) | Study design | Study Population | Administration language of instrument | Methods used | Response rate |
| --- | --- | --- | --- | --- | --- | --- |
| Bonevski et al (2010)  Australia | To compare reliability of computer and paper methods of assessing self-reported alcohol use with alcohol urine assay measurement used as a reference standard. | Longitudinal study. A randomised cross-over design was used with 4-7 days between time 1 and time 2. | Group 1 was 30% male and 70% female, Group 2 37% male and 63% female, Group 3 44% male and 56% female and Group 4 41% male and 59% female. Group 1 mean age 25 years. Group 2 mean age 27 years. Group 3 mean age 25 years. Group 4 mean age 25 years. Subjects were randomly assigned to one of 4 groups and completed the survey at 2 time points. Group 1 completed a computer administered version of the survey at both times, group 2 completed a computer survey at time 1 and paper survey at time 2, group 3 completed a paper survey at time 1 and computer survey at time 2 and group 4 completed a paper administered version of the survey at both times. | English | Participants were asked to recall alcohol intake using either a computer or paper administered measure. 4-7 days later both modes of measures were administered again. At Time 1 all participants also completed alcohol consumption (ethyl alcohol urine assay). | 76% response rate. |
| Chaikelson et al (1994)  Canada | To assess the reliability and validity of the Concordia Lifetime Drinking Questionnaire  (CLDQ), a self-report short-term recall measure. | Longitudinal study. Data was taken from 2 time points (1987 and 1990). | Random sampling was used. The sample was 100% male with mean age 69 years. Elderly male Canadians were administered the questionnaire via structured face to face interview. Wives also asked same questions via written questionnaire to assess concordance. | English or French | CLDQ results compared to alcohol test the MAST (Michigan Alcoholism Screening Test [55]) for reliability and validity. | 22% response rate. |
| Crum et al (2002)  USA | To test the hypothesis that there is greater self-reporting of alcohol consumption using a quantity-frequency questionnaire compared to an interview on usual alcohol consumption. | Longitudinal study. Data was obtained from a single time point, the 1993-1994 follow-up. | Random sampling was used. The sample was 58% female and 42% male with mean age 76.2 years. Data was obtained from the 1993-1994 follow-up of the Washington County cohort of men and women 65 years and older who participated in the Cardiovascular Health Study. | English | Participants completed a measure of their usual alcohol consumption in two ways: (1) a quantity-frequency measure; (2) same questions asked in an interview about drinking habits. | 75% response rate. |
| Cutler et al (1988)  UK | To assess alcohol consumption measured using a quantity-frequency measure and the CAGE test (Cut down, Annoyed, Guilty, Eye-opener (test for problem alcohol use [45])) for problem drinking. | Cross-sectional sample of respondents who returned the Health Survey Questionnaire conducted annually. | Random sampling was used. 63.4% of the sample were male and 36.6% female. No median or mean age was reported but participants were aged 18 and older. Participants were invited to attend a face to face interview, where weekly alcohol consumption was estimated using a quantity-frequency scale and alcohol consumption for the week preceding the interview was measured. | English | CAGE responses and the quantity-frequency questions taken from Health Survey Questionnaire were compared. | 61.2% response rate. |
| Dollinger et al (2009)  USA | To test the reliability and validity of a quantity-frequency measure. | Longitudinal study. Undergraduate college students reported on their alcohol consumption on two occasions 11 days apart. Also reported on hours spent studying, socialising and religious behaviours, logging daily over 2 weeks. | The sample was composed of volunteers and was 61% female and 39% male with a mean age 22 years. On 2 occasions college students reported on the frequency and quantity of alcohol consumption, and recorded their intake nightly over 2 weeks. | English | Responses to quantity-frequency measures at both time points compared. Nightly log of alcohol consumption compared to hours spent studying, socialising and religious behaviours. | 70.7% response rate. |
| Greenfield et al (2014)  USA | To assess the test-retest reliability of retrospective lifetime drinking measures and the predictive validity of lifetime heavy drinking (days 5+ drinks) in teens, 20s and 30s for incidence of alcohol use disorders. | Longitudinal study. Data was taken from 2 time points using a subset of respondents from the 2005 US National Alcohol Survey with a 2.7 year mean delay between measures. | Random sampling was used. Respondents were 48.1% male and 53.2% female and aged over 18 years. Data was taken from 2 time points using a subset of respondents from the 2005 US National Alcohol Survey. | English | Participants completed questionnaires and a follow-up survey by phone or mail. | 93.5% response rate. |
| Gruenewald et al (1995)  USA | Measurement of test-retest reliability of a graduated-frequency measure. | Longitudinal study. Data was taken from a population survey of 96 Californian zip codes with 1 month between measure applications. | Random sampling was used. Respondents were 43.5% male and 56.5% female and aged 18 years or older. Participants completed a telephone survey on 2 occasions assessing alcohol intake. | English | Responses to graduated-frequency measures at two time points compared. | 65% response rate. |
| Hansell et al (2008)  Australia | To compare diagnoses of alcohol dependence using DSM-IIIR (Diagnostic and Statistical Manual of Mental Disorders 3rd edition) and DSM-IV (Diagnostic and Statistical Manual of Mental Disorders 4th edition) criteria with alcohol consumption measured using a quantity-frequency measure of annual consumption. | Longitudinal study conducted in three different phases totalling 13 years. | Random sampling was used. Respondents were 40% male and 60% female and aged between 19-90 years old. Data was collected in telephone interviews conducted in three collection phases. Phase 1 data were collected from 1992-1993, phase 2 data from 1996-2000 and phase 3 from 2001-2005. | English | The measures examined were a dependence score, based on DSM-IIIR and DSM-IV criteria for substance dependence, and a quantity × frequency of alcohol consumed taken from the quantity-frequency measure. | 25% response rate |
| Hilton (1989)  USA | To compare self-report alcohol consumption from daily alcohol intake diaries to a retrospective recall of alcohol consumption. | Longitudinal study. Volunteer participants kept a 10 week record of their alcohol intake which was followed up by a 30 day graduated-frequency and 2 week beverage specific quantity-frequency measure. | Volunteer sample. Respondents were 50% male and 50% female and had a mean age of 30 years. The volunteer participants were recruited from the San Francisco Bay Area newspaper. | English | Participants completed 2 retrospective recall measures-graduated-frequency and beverage-specific quantity-frequency measures post diary completion. Responses compared. | 84% response rate. |
| Koppes et al (2002)  Netherlands | To compare the quantity-frequency measure with a dietary history interview (which included questions on alcohol intake). | Longitudinal study. Participants were respondents to the Amsterdam Growth and Health Longitudinal Study conducted annually. | Random sampling was used. Respondents were 46% male and 54% female with mean age 36 years. Data was collected from 1 time point, the 2000 follow-up measurement of 171 male and 197 female participants from the Amsterdam Growth and Health Longitudinal Study. | Unclear whether measure was administered in English or Dutch. | Subjects visited study premises for 1 day. The quantity-frequency measure and dietary history interview were based on alcohol consumption over the previous month and were completed in no particular order. | 61% response rate. |
| LaBrie et al (2004)  USA | The purpose of the study was to determine the comparability of group Timeline follow back and individual Timeline follow back. | Cross sectional study. 2 groups of college students completed a 30 day recall of their alcohol intake in either an individual or group format. | The sample was composed of volunteers and was 100% male with a mean age of 20.6 years. 211 male college students participated. | English | Drinking variables assessed were drinking days, average drinks, and total drinks during a 30-day period. | 56% response rate for group format and 44.1% for individual format. |
| Lennox et al (1996)  USA | Examines structural relationships derived from formally modelling latent variables of adverse alcohol-related constructs. | Longitudinal study. Participants recalled their intake for a series of questions covering alcohol abuse, dependence and adverse consequences. | Analysis was conducted of a sample of a household survey aged 18-64 years. Gender proportions were not reported. Responses were analysed from 1 time point (the 1991 follow-up) from 8,755 participants in the 1988 National Household Survey of Drug Abuse. | English | Used a latent variable approach. In this model covariation among multiple indicators was used as an estimate of the latent construct. | 26.7% response rate |
| McGinley et al (2014)  USA | To examine the construct validity of a 30 day graduated-frequency measure of alcohol consumption. | Longitudinal study. Data was collected from participants in the 2010 National Survey of Drug Use and Health, conducted annually. | A sample of 18-20 year olds were selected from respondents to the National Survey on Drug Use and Health. Gender proportions were not reported. | English | Quantity and frequency of alcohol consumption estimates derived from graduated-frequency measure. Estimates compared to the quantity-frequency measure. | 10.3% response rate. |
| Northcote and Livingston (2011)  Australia | To test the validity of a ‘last occasion’ retrospective self-report compared to corresponding field observations of alcohol consumption. | Longitudinal study. Undergraduate university students were asked to recall alcohol consumed 1-2 days after drinking occasion. | Respondents were 47.3% male and 53.3% female and aged 18-25 years. | English | Participants reported number of alcoholic drinks consumed 1-2 days after drinking occasion which was compared to reported alcohol intake observed by peer-based researchers on the occasion. | 54% response rate |
| O’Hare et al (1991)  USA | To compare retrospective recall of alcohol consumption over the previous week with a graduated-frequency measure of typical weekly consumption. | Longitudinal study. Data was collected from a cohort of undergraduate students enrolled in the 1987 Rutgers Student Alcohol and Drug Survey. | Respondents were 41.6% female 58.4% male and with mean age 20.6 years. | English | Participants were asked to complete mailed questionnaire with both measures of alcohol consumption included. | 75.8% response rate |
| O’Hare et al (1997)  USA | To compare the sensitivity, specificity and positive predictive value of a quantity-frequency measure, 7 day retrospective recall of alcohol intake and a modified version of the MAST. | Longitudinal study. Undergraduate college students completed a 7 day recall and quantity-frequency measure of their alcohol consumption over 7 days. | Random sample of an undergraduate university population. Gender proportions were reported as ‘representative of sex’. Respondents had a mean age of 18.7 years. | English | All students completed quantity-frequency questions, MmMAST and 7 day recall. The MmMAST was used as a criterion variable. | 99% response rate. |
| Parker et al (1996)  USA | To investigate whether a question on alcohol intake asked as part of a general health survey could be used to reliably and validly measure alcohol intake. | Longitudinal study. Data was taken from 3 time points over 6 years from a general population survey. | Random sampling was used. Respondents were 39% male and 61% female and aged 18-64. Data was taken from surveys 1987-1989, 1989-1990 and 1992-1993 of the Pawtucket Health Program conducted among home dwelling adults. | English | Alcohol intake assessed with food frequency question as a component of the general health survey was compared against alcohol intake assessed with a graduated-frequency measure as part of a survey. | 60% response rate. |
| Poikolainen et al (2002)  Finland | To compare the accuracy of a quantity-frequency questionnaire and a graduated-frequency questionnaire as methods of obtaining self-reported alcohol intake compared to a daily recall measure and blood alcohol measurement. | Cross-sectional study. Respondents completed questions asking about alcohol intake before and after completing daily recall measure of alcohol intake over 1 month. | Volunteer sample recruited from their workplace. Respondents were 83% female and 17% male with a mean age of 42 years. | English | Quantity-frequency and graduated-frequency obtained before and after 1-month daily recall on alcohol intake. Blood sample obtained at outset. | 65% response rate. |
| Read et al (2006)  USA | To develop and validate a measure designed to capture a range of alcohol-related consequences experienced by male and female college students (compared to a past 90 day recall of alcohol intake). | Longitudinal study. Undergraduate students recalled their alcohol consumption over 90 days and completed alcohol consequences measure. | College students who reported drinking different amounts of alcohol were selected for the sample to be representative of variation in drinking levels. Respondents were 52% female and 48% male with a mean age 19 years. | English | College students completed self-report questionnaire on demographic characteristics, drinking behaviours and drinking consequences. Drinking consequences assessed with composite measure based on Drinker Inventory of Consequences and Young Adult Alcohol Problem Screening Test developed by researchers. | 99.4% response rate. |
| Rehm et al (1999)  Canada | To compare three measures (drinking occasion quantity-frequency measure, annual graduated-frequency measure and past week short-term recall measure) for estimating high risk drinking and alcohol-related harm. | Cross sectional study with a within-participants design conducted from 1990-1994. | The sample was chosen to be representative of the wider drinking population. Respondents were 48% male and 52% female, and chosen to be representative of age ≥18 years. | English | Population samples from 4 surveys conducted for Alcohol Research Group. Surveys used computer-assisted telephone interviews with random digit dialling sampling techniques. | 61% response rate. |
| Reid et al (2003)  USA | To compare a weekly quantity-frequency measure, CAGE test, AUDIT test (Alcohol Use Disorders Identification Test [44]), heavy drinking measure and binge drinking measure to self-report alcohol intake among older veterans. | Cross sectional study with 5 different methods of measuring alcohol consumption applied over 6 months to 2 population samples. | Random sampling was used. The veteran primary care sample was 3% female 97% male and the community dwelling sample was 60% female 40% male. Mean ages were 73.1 for the veteran primary care sample and 75.9 for the community dwelling sample. | English | Telephone call allowed self-report of quantity-frequency measure, binge and heavy drinking questions, and the AUDIT and CAGE tests. | 81% response rate. |
| Russell et al (1991)  USA | To assess the reliability and validity of a beverage-specific quantity-frequency measure. | Longitudinal study involving respondents of the 1986 New York Statewide Survey. | Random sampling was used. Respondents were 50.5% male and 49.5% female and aged over 18 years. Data was taken from 1 time point of the survey. | English | Quantity-frequency questions were asked about the amount and frequency of particular alcoholic beverages consumed via telephone interview using a random-digit-dial technique and supplemented by samples of homeless people, college students and those without telephones. | 66% response rate. |
| Sander et al (1997)  USA | To investigate bias and concurrent validity of traumatic brain injury patients’ self-report of alcohol consumption by examining concordance with relatives’ reports. | Cross-sectional study which measured alcohol use 1 year post injury alongside their relatives’ reports. | 175 patients with traumatic brain injury were recruited from a medical rehabilitation centre along with their relatives. Respondents were 65% male and 35% female. Mean age 39.2 years for patients and 45.9 years for relatives. | English | Alcohol use examined 1 year after injury through quantity-frequency measure and brief MAST test. Patients and their relatives both completed measures and concordance between reports were examined. | 88% response rate. |
| Searles et al (1995)  USA | To test the reliability and validity of recalling daily alcohol intake. | Longitudinal study involving recall of alcohol intake over 112 days using adults enrolled in Vermont Alcohol Research Centre. | The sample was chosen to be representative of male drinking population in Vermont enrolled in the Alcohol Research Centre. Respondents had a median age of 28 years (ranging from 21-56 years) and were 100% male. | English | Subjects self-reported daily alcohol intake via telephone. At 90 days subjects completed an interview using DSM (Diagnostic and Statistical Manual of Mental Disorders [54]) criteria to assess alcohol abuse or dependence. | 93% response rate. |
| Searles et al (2000)  USA | To compare daily alcohol consumption via self-report over 366 consecutive days with a retrospective report of alcohol consumption. | Longitudinal study involving 366 day recall from a sample of those participating in research at the Vermont alcohol research centre. | Volunteer sample of those enrolled in the Vermont Alcohol Research Centre. Respondents were 100% male and had a mean age of 36.2 years for those without alcohol problems tested at outset and 30.4 years for those with alcohol problems. | English | Participants recorded alcohol intake on interactive voice response system using telephones. In person interviews were conducted every 13 weeks during which they completed timeline follow back. Results were compared. | 48% response rate. |
| Tuunanen et al (2013)  Finland | To test the hypothesis that more alcohol intake will be self-reported using a quantity-frequency measure compared to recall of alcohol consumed over the past week. | Cross sectional study with data taken from 1 mailed questionnaire. | The sample included 45 year olds resident in Finnish city of Tampere. The sample was 100% male. | Unclear whether measure was administered in English or Finnish. | Participants completed a mailed health questionnaire which invited previous week recall of alcohol intake, a quantity-frequency measure and structured quantity-frequency questions based on the Alcohol Use Disorders Identification Test [44]. | 55.1% response rate. |
| Weingardt et al (1998)  USA | To investigate the concurrent and predictive validity of self-report alcohol consumption measures (peak monthly, typical weekend and daily). | Longitudinal study with data taken from 2 time points over 4 years. | Random sampling was used. Respondents were 58% female and 42% male and aged 18-20 years. Data was taken from 1990 and 1994 cohorts of college undergraduate students. | English | Peak consumption, typical weekend quantity and typical daily quantity measures used to derive binge drinking data to analyse validity. Binge drinking defined as 5-6 drinks per occasion for men and 3-4 drinks per occasion for women. | 76.6% response rate. |
| Whitfield et al (2004)  Australia | To assess test-retest reliability of an annual quantity-frequency measure compared with past week recall. | Longitudinal study. Data taken from 3 waves conducted over 15 years of the Australian Twin Registry. | Voluntary sample. Respondents were 36% male and 64% female with a mean age of 33.7 years. Data was taken from 3 waves (1980, 1989 and 1993) using adult male and female participants of the Australian Twin Registry. | English | Test-retest reliability was calculated as correlations between occasions and between measures. Relationships between alcohol use and lifetime DSMIIIR (Diagnostic and Statistical Manual of Mental Disorders revised 3rd edition) alcohol dependence examined. | 69.7% response rate. |

Table Legend: Table presenting the characteristics of each included study grouped by authors, aims, study design, study population, administration language of instrument, methods used and response rate
