# Supplementary material for: Evaluation of the psychometric properties of self-reported measures of alcohol consumption: a COSMIN systematic review
Source: Subst Abuse Treat Prev Policy. 2018 Feb 2;13:6. doi: 10.1186/s13011-018-0143-8 (PMC5797334; doi:10.1186/s13011-018-0143-8)
Supplement: Supplementary file 3 — Psychometric properties of included studies grouped into results reported by study authors and COSMIN quality ratings assigned by review authors (n = 28). (DOCX 41 kb) [file 13011_2018_143_MOESM3_ESM.docx]

Additional file 3 Table S2 Psychometric properties of included studies

|  | Studies and measures | Properties | Study results | Methodological quality assessment scores |
| --- | --- | --- | --- | --- |
| Quantity-frequency | Bonevski et al (2010)  Weekly quantity-frequency measure. A two-item quantity/frequency measure of alcohol consumption was used. Patients were asked “How many days in an average week do you usually drink alcohol?” and “On a day when you drink alcohol, how many standard drinks do you usually have?” | Test-retest reliability | Test-retest reliability  Kappa coefficients for groups who undertook quantity-frequency measure through computer or paper administration and urinalysis results at time points 1 and 2, ranged between 0.90 and 0.96. The group who completed two computer administered measures reported a coefficient of 0.93, the group who completed two paper administered measures reported a coefficient of 0.90, the group who completed a computer administered measure at time 1 and paper administered measure at time 2 reported a coefficient of 0.96 and the group who completed a paper administered measure at time 1 and computer administered measure at time 2 reported a coefficient of 0.92. Test-retest reliability was good. | Test-retest reliability (poor) |
|  | Crum et al (2002)  Weekly quantity-frequency measure. Responses were converted into grams of ethanol per day.  Short-term recall measure (past week recall). Alcohol intake was derived from responses to an interview with the questions contained within a 62-item medical and personal history questionnaire.  The questions in both questionnaires sought details of alcoholic beverages type (12 ounce can/bottle of beer, 6 ounce glass of wine, shot of liquor) together with the frequency each beverage was consumed (daily, weekly, monthly, yearly or never). | Hypothesis validity  Inter-rater reliability | Hypothesis validity  It was hypothesised that there would be greater disclosure in self-reports of alcohol intake using the quantity-frequency measure over the past week recall measure. Past week recall of alcohol intake was 15-20% lower than the quantity-frequency measure indicating good hypothesis validity.  Inter-rater reliability  The kappa value between the short-term recall and quantity-frequency measure was 0.76 indicating good inter-rater reliability. | Hypothesis validity (good)  Inter-rater reliability (poor) |
|  | Cutler et al (1988)^a^  Weekly quantity-frequency measure. Alcohol consumption was assessed using a quantity-frequency scale and includes the CAGE questions and a question on whether respondents think they have an alcohol problem. Excessive drinkers were defined as men whose weekly consumption by this measure was not less than 35 units per week and for women at least 21 units per week. | Criterion validity | Criterion validity  Using recall of alcohol consumed over the past week (a short-term recall measure) as a criterion standard. Respondents were classified into gender groups and excessive and non-excessive alcohol drinkers. The quantity-frequency measure compared to past week recall reported p values ranging from <0.001 for all non-excessive drinkers and excessive female drinkers. A p value of <0.05 was reported for excessive male drinkers. Ability of the quantity-frequency measure to predict excessive alcohol consumers reported sensitivity (42.9) specificity (97.1) positive predictive value (65.8) negative predictive value (92.8) for males and sensitivity (46.6) specificity (98.6) positive predictive value (50.3) negative predictive value (98.4) for females indicating good criterion validity | Criterion validity (excellent) |
|  | Hansell et al (2008)  Annual quantity-frequency measure. Respondents were asked to recall typical number of drinks on drinking days. Responses were coded into categories ‘0 drinks, 1-2 drinks, 3-4 drinks, 5-6 drinks, 7-8 drinks, 9-11 drinks, 12-15 drinks, 16-18 drinks, 19-24 drinks, 25-30 drinks or 31 or more drinks’. | Test-retest reliability | Test-retest reliability  When examined as continuous data quantity x frequency of alcohol consumed was 0.61 between phase 1 and phase 3, and 0.55 between phase 2 and phase 3. When examined as categorical data quantity x frequency of alcohol consumed was 0.64 between phase 1 and phase 3, and 0.59 between phase 2 and phase 3. The quantity-frequency measure has moderate test-retest reliability. | Test-retest reliability (poor) |
|  | Hilton (1989)  Short-term recall measure (10 week recall). Measure broken up into 10 weekly instalments mailed to participants.  Graduated-frequency measure (30 day recall). Contained questions asking how many days out of 30 respondent drank in amount ranges 1-2 drinks, 3-4 drinks, 5-7 drinks, 8-11 drinks, 12 or more drinks.  Beverage specific Quantity-frequency measure (2 week recall). Questions included days drinking beer, wine or liquor respectively, and they amount of ounces they estimate they consumed. | Hypothesis validity (convergent validity) | Convergent validity  Correlations between mean recall calculated from the 10 week recall and 2 week recall using quantity-frequency measure were positive at; 0.88 for volume of drinks consumed, 0.85 for days of beer consumed, 0.89 for days of beer usually consumed, 0.80 for days of wine consumed, 0.66 for days of wine usually consumed, 0.81 for days of liquor consumed and 0.65 for days of liquor usually consumed. Overall the quantity-frequency measure had moderate to good convergent validity with the short-term recall measure. | Convergent validity (fair) |
|  | Koppes et al (2002)^a^  Quantity-frequency measure (ranging from never drinking to daily alcohol intake). Respondents were asked how often they consumed 1 or more alcoholic drinks. Frequency options were never, monthly or less often, two or four times per month, two or three times per week, or four times per week or more often. Respondents were then asked how many alcoholic drinks (glasses) they consumed on an average drinking day. Quantity options were 1, 2, 3, 4-5, 6-8 or 9 or more.  Short-term recall measure (dietary history interview). A trainer interviewer asked respondents what, how often and how much they ate and drank (including alcohol consumed) during each day. | Criterion (concurrent validity) | Criterion validity (concurrent validity)  The dietary history interview was reported as a criterion standard. Correlation coefficients between the two measures were reported to be 0.77 for men and 0.87 for women. Good correlation was reported between the quantity-frequency and dietary history interview short-term recall measures which indicates good concurrent validity. | Criterion validity (poor) |
|  | Lennox et al (1996)  Quantity-frequency measure of alcohol consumption over past 30 days. Respondents were asked about number of drinking days, how many drinks consumed on drinking days, number of days with 5 or more drinks and highest amount drank on one occasion. | Structural validity | Structural validity  Using confirmatory factor analysis: alcohol abuse and alcohol dependence between constructs correlates at 0.36, alcohol abuse and alcohol consequences between constructs correlates at 0.28. No evidence of any correlation between alcohol consequences, dependence or alcohol abuse using a quantity-frequency measure of consumption showing poor structural validity. | Structural validity (fair) |
|  | Poikolainen et al (2002)  Annual quantity-frequency questionnaire. This involved 10 drinking frequency options (never, 1-2 times per year, 3-4 times per year, approximately every second month, approximately once a month, approximately twice a month, once a week, 2-3 times weekly, 4-5 times weekly, 6-7 times weekly). Quantity questions were beverage specific per occasion with highest options including 5 or more litres of beer, 1.5 litres of wine or 1 litre of more of spirits.  Daily graduated-frequency measure. Responses ranged from 15 or more drinks per day to 1-2 drinks per day, with 1 drink described as a standard serving with an alcohol content of 12g each.  Short-term recall measure (past month recall of intake). This was a daily recall of alcohol intake. | Test-retest reliability Hypothesis validity (convergent validity) | Test-retest reliability Test-retest reliability was measured as a coefficient between the quantity-frequency measure administered before and after application of the short-term recall measure. Correlation coefficient between time 1 and 2 was 0.93 for the quantity-frequency measure, indicating good test-retest reliability.  Convergent validity. Coefficients between the quantity-frequency measure at time points 1 and 2 and the graduated-frequency measure at time point 1 were 0.88 and 0.90. Coefficients between the quantity-frequency measure at time point 1 and 2 and the graduated-frequency measure at time point 2 were 0.91 and 0.96. Coefficients between the quantity-frequency measures at time point 1 and 2 and the short-term recall measure were 0.95 each indicating good convergent validity between the measures. | Test-retest reliability (good)  Convergent validity (good) |
|  | Rehm et al (1999)  Quantity-frequency measure for drinking occasion. Respondents were asked about their usual number of drinks consumed per drinking occasion.  Annual Graduated-frequency measure. Respondents were asked to recall their highest number of drinks for any occasion in the past year, with the highest quantity item 12 or more drinks.  Short-term recall measure (past week recall). Participants were asked for the number of drinks consumed on each of the seven days before the survey, beginning with the most recent day. | Hypothesis validity (convergent validity and predictive validity) | Convergent validity Correlations between quantity-frequency measure and graduated-frequency measure were 0.76 and 0.73 for males and females respectively. Correlations with the quantity-frequency measure and weekly drinking recall was around 0.40 indicating moderate to good convergent validity for the measure.  Predictive validity  The quantity-frequency yields estimates for mortality that are 13% higher. Years of life lost (measure of mortality) for individuals measured using quantity-frequency methods were 9% higher than weekly drinking recall indicating poor predictive validity. | Convergent validity (fair)  Predictive validity (excellent) |
|  | Reid et al (2003)  Weekly quantity-frequency measure. Participants were asked to recall average number of drinking days per week, number of drinks consumed per occasion and types of beverages consumed by current drinkers. Individuals who consumed more than 14 drinks per week were defined as heavy drinkers. | Inter-rater Reliability | Inter-rater reliability  For population sample 1 kappa values were 0.44 for a measure of consumption of ≥5 drinks per occasion and 0.33 for a measure of drinking ≥14 drinks per week over 50 or more years. For population sample 2 kappa values were 0.21 for a measure of consumption of ≥5 drinks per occasion and 0.46 for a measure of drinking ≥14 drinks per week over 50 or more years. Moderate to poor inter-rater reliability was reported between the quantity-frequency measure, a measure of binge drinking and a measure of heavy cumulative drinking. | Inter-rater Reliability (fair) |
|  | Russell et al (1991)^a^  Typical annual beverage-specific Quantity-frequency measure. Average absolute alcohol was calculated from frequency items (ranging from 3 times daily to more to less than once a month) and quantity (ranging from 1 drink to 7 drinks or more). This was completed for beer, wine and liquor for respondents. | Criterion validity | Criterion validity  Correlations between the beverage-specific quantity-frequency measure and favoured beverage frequency (the criterion standard) were between 0.73 and 0.77 for subtypes of alcohol reported. Beverage-specific quantity-frequency measures show good criterion validity when compared against a favoured beverage frequency question which acted as a criterion standard. | Criterion validity (poor) |
|  | Sander et al (1997)^a^  Annual quantity-frequency measure. Questions on quantity and frequency of alcohol consumed over the past year were asked with participants classified as abstinent, infrequent, light, moderate or heavy drinkers. | Criterion validity (concurrent validity) | Concurrent validity  Concordance between patient and relative report (the criterion standard) using a quantity-frequency measure showed 95.4% agreement. High level of agreement found between patient and relative reports of alcohol consumption using a quantity-frequency measure, indicating good criterion validity for the measure. | Criterion validity (fair) |
|  | Tuunanen et al (2013)  Quantity-frequency measure (typical drinks consumed per occasion). Participants asked how often they drank alcohol and how many drinks per drinking occasion. Reported drink numbers were translated into grams of absolute alcohol.  Short-term recall measure (past week recall). Respondents asked how many glasses or bottles of drinks containing alcohol they consume per week. | Hypothesis validity | Hypothesis validity  The hypothesis tested was that there would be greater alcohol consumption reported using a quantity-frequency question over mean weekly intake. Mean weekly intake gave lower values of weekly alcohol consumption. The quantity-frequency measure reported mean alcohol consumption (g of absolute alcohol) for all, moderate, risky and heavy episodic drinkers as 170.1, 102.4, 461.3 and 319.4g, higher than the short-term recall measure which indicated good hypothesis validity. | Hypothesis validity (fair) |
|  | Whitfield et al (2004)  Annual quantity-frequency measure. Participants were asked about their frequency of alcoholic drink consumption in the past year along with usual number of drinks taken.  Short-term recall measure (past week recall of alcohol intake). Respondents completed a table indicating how many glasses of beer, wine, spirits, sherry and other alcoholic drinks they had consumed in the past week. Glass volumes were quoted for particular drinks for both measure types to standardise quantities of alcohol reported. | Test-retest reliability | Test-retest reliability  Test-retest reliability was calculated as correlations between administrations (years 1980-1993). Test-retest reliabilities for the quantity-frequency measures from surveys were 0.60 (surveys between 1980 and 1989) 0.56 (between 1980 and 1993) 0.57 (between 1989 and 1980) 0.69 (between 1989 and 1993) 0.54 (between 1993 and 1980). Moderate to good test-retest reliabilities were reported for the annual quantity-frequency measure. | Test-retest reliability (fair) |
| Graduated-frequency measure | Dollinger et al (2009)  Daily graduated-frequency measure. “How often do you consume alcoholic beverages?” recorded on an 8-step scale ranging from 0 (never), through 4 (2 days per week), to 7 (just about every day); Responses to “How much alcohol do you typically consume per drinking occasion?”—defining 1 drink as 1 can of beer, glass of wine, mixed drink, or shot—were recorded on a 6-step scale ranging from 0 (I do not drink) to 5 (more than 6 drinks per occasion).  Short-term recall measure (daily alcohol intake recall). Participants were asked to recall their intake through a daily log to be completed each night. | Test-retest reliability  Hypothesis validity (divergent and convergent validity ) | Test-retest reliability  The graduated-frequency measure reported an alcohol quantity coefficient of 0.85 and an alcohol frequency coefficient of 0.84 indicating good test-retest reliability.  Divergent validity  Religion-by-alcohol correlations were negative with values from -0.14 to -0.37.  Convergent validity. Hours spent socialising and hours of study had positive correlations with alcohol with values of 0.40 and 0.41 respectively.  Good divergent and convergent validity were reported. | Test-retest reliability (fair)  Divergent validity (fair)  Convergent validity (fair) |
|  | Gruenewald et al (1995)  Monthly graduated-frequency measure. Frequent drinkers were asked about alcohol intake in the past 28 days or less frequent drinkers were asked about alcohol intake in the past year which was scaled to per month. A drink was defined as a 12-oz of beer, a 5-oz glass of wine or a 1-oz shot of distilled spirits. | Test-retest reliability | Test-retest reliability  Reported as coefficients for average drinking quantity r=0.76 and for variance in drinking quantities r=0.78. Test-retest reliability coefficients for drinking frequency highest at r=0.85. The graduated-frequency measure shows good test-retest reliability. | Test-retest reliability (fair) |
|  | Hilton (1989)  Short-term recall measure (10 week recall). Measure broken up into 10 weekly instalments mailed to participants.  Graduated-frequency measure (30 day recall). Contained questions asking how many days out of 30 respondent drank in amount ranges 1-2 drinks, 3-4 drinks, 5-7 drinks, 8-11 drinks, 12 or more drinks.  Beverage specific Quantity-frequency measure (2 week recall). Questions included days drinking beer, wine or liquor respectively, and they amount of ounces they estimate they consumed. | Hypothesis validity (convergent validity) | Convergent validity Calculated using mean recall from both a 10 week short-term recall and 30 day graduated-frequency measure. Correlations ranged from; volume of drinks correlation 0.89, frequency of alcohol consumed 0.68, 12 or more drinks consumed 0.63, days 8-11 drinks consumed 0.69, days 5-7 drinks consumed 0.59, days 3-4 drinks consumed 0.50, days 1-2 drinks consumed 0.61. Convergent validity was moderate to good. | Convergent validity (fair) |
|  | McGinley et al (2014)  Graduated-frequency measure of alcohol consumption over past 30 days. Participants were asked the same questions with frequency item responses ranging from 0.-30 and quantity item responses ranging from 0-20. | Construct validity | Construct validity  Mid values were calculated for number of drinking days which ranged from 10-19 days. The mid values for quantity of alcohol consumed were (3.5) and (14.5) for frequency which were not an accurate measure of central tendency of the data indicating poor construct validity. | Construct validity (fair) |
|  | O’Hare et al (1991)  Weekly graduated-frequency measure. Respondents were asked how often they usually drank alcoholic beverages, with responses ranging from never to 7 or more times per week. 1-2 drinks, 3-4 drinks, 5-7 drinks, 8-10 drinks, 11-15 drinks or 16 or more drinks were the quantity items.  Short-term recall measure (retrospective recall of past 7 day alcohol intake). Respondents were asked to fill in boxes provided for each of the previous 7 days with the number of drinks consumed on each day. | Hypothesis validity (convergent validity) | Convergent validity Participants reported higher levels of drinking using the retrospective recall than a typical weekly graduated-frequency measure. Correlations between the graduated-frequency and retrospective recall measures were significant at 0.74, with gender specific correlations for men as 0.79 and women 0.60. Moderate to good convergent validity was reported. | Convergent validity (good) |
|  | O’Hare et al (1997)^a^  Weekly graduated-frequency measure. Respondents were asked how often they drank ranging from never to seven or more times per week. Quantity items ranged from 0 drinks to 16 or greater per occasion.  Short-term recall measure (retrospective recall of past 7 day alcohol intake). Starting with the previous day respondents were asked to fill in the number of drinks they consumed over the past 7 days. | Criterion validity  Hypothesis validity (predictive validity) | Criterion validity  The association between the graduated-frequency measure and MAST was significant (p<0.01) indicating good criterion validity.  Predictive validity Using MAST cut-off point ≥3 as a criterion variable sensitivity and specificity of the measure was 75 and 66. Using MAST cut-off point ≥2 sensitivity and specificity of the measure was 69 and 81.8. Good predictive validity was reported. | Criterion validity (fair)  Predictive validity (fair) |
|  | Parker et al (1996)^a^  Short-term recall measure (beverage specific past 24 hour recall). Participants were asked whether they had any alcohol to drink in the last 24 hours such as wine, beer or any type of liquor  Annual graduated-frequency measure. Respondents were asked how often in the past year they had consumed each type of alcoholic beverage. Options ranged from never or less than once per month to 6 or more times per day. | Criterion validity (concurrent validity)  Inter-rater Reliability | Concurrent Validity  The short-term recall measure acted as the criterion standard. Correlations were found between both measures ranging from 0.08 (p<0.001), 0.38 (p<0.001) and 0.81 (p<0.001) for low, medium and high consumers of alcohol respectively. Poor concurrent validity was reported between measures when people were classified as low or moderate drinkers, but good concurrent validity was reported for high consumers of alcohol.  Inter-rater reliability  Kappa values between measures were 0.28 for those who consumed any alcohol type, 0.38 for beer, 0.44 for liquor and 0.37 for wine.  Inter-rater reliability was poor (below 0.70). | Criterion validity (poor)  Inter-rater Reliability (fair) |
|  | Poikolainen et al (2002)  Annual quantity-frequency measure. This involved 10 drinking frequency options (never, 1-2 times per year, 3-4 times per year, approximately every second month, approximately once a month, approximately twice a month, once a week, 2-3 times weekly, 4-5 times weekly, 6-7 times weekly). Quantity questions were beverage specific per occasion with highest options including 5 or more litres of beer, 1.5 litres of wine or 1 litre of more of spirits.  Daily graduated-frequency measure. Responses ranged from 15 or more drinks per day to 1-2 drinks per day, with 1 drink described as a standard serving with an alcohol content of 12g each.  Short-term recall measure (past month recall of intake). This was a daily recall of alcohol intake. | Test-retest reliability Hypothesis validity (convergent validity) | Test-retest reliability Test-retest reliability was assessed by a correlation coefficient between graduated-frequency measures administered at two time points before and after a 1 month drinking diary. The correlation coefficient was 0.90, indicating good test-retest reliability.  Convergent validity.  Coefficients between the graduated-frequency measures at time points 1 and 2 and the quantity-frequency measure at time point 1 were 0.88 and 0.91. Coefficients between the graduated-frequency measures at time points 1 and 2 and the quantity-frequency measure at time point 2 were 0.96 and 0.90. Coefficients between the graduated-frequency measures at time points 1 and 2 and the short-term recall measure were 0.90 and 0.93, indicating good convergent validity between these measures. | Test-retest reliability (good)  Convergent validity (good) |
|  | Rehm et al (1999)  Quantity-frequency measure for typical drinking occasion. Respondents were asked about their usual number of drinks consumed per drinking occasion.  Annual graduated-frequency measure. Respondents were asked to recall their highest number of drinks for any occasion in the past year, with the highest quantity item 12 or more drinks.  Short-term recall measure (past week recall). Participants were asked for the number of drinks consumed on each of the seven days before the survey, beginning with the most recent day. | Hypothesis validity (convergent validity and predictive validity) | Convergent validity Correlations between quantity-frequency measure and graduated-frequency measure were 0.76 and 0.73 for males and females respectively. Correlations with both measures and weekly drinking recall were both around 0.40 indicating moderate to good convergent validity.  Predictive validity  Alcohol-related hospital discharges (measure of morbidity) estimated from graduated-frequency measures are 12% higher than those estimated from weekly drinking recall. The graduated-frequency measure yields estimates for mortality that are 22% higher than weekly recall. Years of life lost (measure of mortality) for individuals measured using graduated-frequency methods were 15% higher than weekly drinking recall. Estimates of productivity losses were 14% higher when measured using graduated-frequency methods over weekly drinking recall indicating good predictive validity. | Convergent validity (fair)  Predictive validity (excellent) |
|  | Weingardt et al (1998)^a^  Graduated-frequency measure (peak monthly alcohol consumption). Respondents asked about occasion of highest alcohol consumption in the past month. Response options for drinkers were 1-2 drinks, 3-4 drinks, 5-6 drinks, 7-8 drinks and 8 or more drinks.  Graduated-frequency measure (typical weekend quantity). Respondents were asked about their alcohol intake over a typical weekend evening during the previous month. Response options remained the same.  Short-term recall measure (typical daily quantity). Respondents asked to fill in number of drinks usually consumed for each day of the week focusing on the past month. | Criterion validity (concurrent validity)  Hypothesis validity (predictive validity) | Concurrent validity  The peak consumption graduated-frequency measure correlated with Rutgers Alcohol Problems Inventory with r value 0.60 and with Alcohol Dependence Scale r value 0.71. The typical weekend quantity graduated-frequency measure correlated with Rutgers Alcohol Problems Inventory with r value 0.58 and Alcohol Dependence Scale with r value 0.59 indicating moderate criterion validity.  Predictive Validity  The peak monthly alcohol consumption graduated-frequency measure classified 18.5% of drinkers as being chronic or on an increased trajectory. The typical weekend quantity graduated-frequency measure classified 4.9% of drinkers as being chronic or on an increased trajectory indicating poor predictive validity. | Criterion validity (good)  Predictive validity (good) |
| Short-term recall measures | Chaikelson et al (1994)^a^  Short-term recall measure (drinking occasions in the previous month recall). This measure established the date of the most recent drink and asks questions about consumption for the month prior to the most recent drink. The number of occasions of use and the quantity per occasion are determined separately for beer, wine and distilled spirits. The interviewer converts the alcohol consumption into standard drinks per week, using a formula  where one standard drink equals 12 ounces of Canadian beer,  5 ounces of wine, 3 ounces of fortified wine, or 1.5 ounces of spirits, each containing approximately 13.6 g of absolute alcohol. | Test-retest reliability  Criterion validity (concurrent validity)  Construct validity | Test-retest reliability  Between 1987 and 1990 kappa coefficients were 0.76 for total lifetime drinking, 0.84 for last reported month and 0.77 for monthly alcohol consumption indicating good test-retest reliability.  Concurrent validity Reported as correlations between self-report and report of spouse which were 0.87 for husband alcohol intake and 0.85 for wife alcohol intake indicating good criterion validity.  Construct validity  Reported as correlations with the MAST self-report test of alcohol intake in 1987 which scored 0.60 with total lifetime drinking and 0.05 with current drinking. Correlations with the 1990 data showed 0.53 with total lifetime drinking and -0.14 with current drinking. Construct validity shows moderate reported correlation with results obtained using MAST. | Test-retest reliability (fair)  Criterion validity (poor)  Construct validity (poor) |
|  | Crum et al (2002)  Weekly quantity-frequency measure. Responses were converted into grams of ethanol per day.  Short-term recall measure (past week recall). Alcohol intake was derived from responses to a questionnaire delivered by interview with the questions contained within a 62-item medical and personal history questionnaire.  The questions in both questionnaires sought details of alcoholic beverages type (12 ounce can/bottle of beer, 6 ounce glass of wine, shot of liquor) together with the frequency each beverage was consumed (daily, weekly, monthly, yearly or never). | Hypothesis validity  Inter-rater reliability | Hypothesis validity  It was hypothesised that there would be greater disclosure in self-reports of alcohol intake using the quantity-frequency measure over the past week recall measure. Past week recall of alcohol intake was 15-20% lower than the quantity-frequency measure. Hypothesis validity was good.  Inter-rater reliability  Inter-rater reliability between both measures in classifying respondents according to alcohol intake was good with kappa statistic value 0.76. | Hypothesis validity (good)  Inter-rater reliability (poor) |
|  | Dollinger et al (2009)  Daily graduated-frequency measure. “How often do you consume alcoholic beverages?” recorded on an 8-step scale ranging from 0 (never), through 4 (2 days per week), to 7 (just about every day); Responses to “How much alcohol do you typically consume per drinking occasion?”—defining 1 drink as 1 can of beer, glass of wine, mixed drink, or shot—were recorded on a 6-step scale ranging from 0 (I do not drink) to 5 (more than 6 drinks per occasion).  Short-term recall measure (daily alcohol intake recall). Participants were asked to recall their intake through a daily log to be completed each night. | Hypothesis validity (divergent and convergent validity ) | Divergent validity Religion-by-alcohol correlations were negative with values from -0.14 to -0.37 indicating good divergent validity.  Convergent validity Hours spent socialising and hours of study had positive correlations with alcohol with values of 0.40 and 0.41 respectively indicating good convergent validity. | Divergent validity (fair)  Convergent validity (fair) |
|  | Greenfield et al (2014)  Short-term recall measure (occasions of ≥5 drinks during specific life decades). Three items assessed the frequency of drinking five or more (5+) drinks on one occasion during specific life decades, i.e., teens, 20's and 30's, using 5 response options: “every day or nearly every day”, “at least once a week”, “at least once a month”, at least once a year”, and “never”. | Test-retest reliability  Hypothesis validity (predictive validity) | Test-retest reliability  Kappa values were calculated for men (0.64) women (0.80), age groups 18-29 (0.59) 30-39 (0.83) 40-49 (0.75) 50-59 (0.74) 60 and older (0.67), white ethnicity (0.71) black ethnicity (0.70) Hispanic ethnicity (0.73), phone interview mode (0.73) mail interview mode (0.72) and those with childhood victimisation (0.75) and those without (0.73) indicating moderate to good test-retest reliability.  Predictive validity Using logistic regression each 780 day disclosure of prior heavy drinking increased risk for alcohol dependence by 18%, increased risk of 2 or more consequences by 21% (by 15% when age of onset was controlled), increased risk for alcohol-use disorder by 18% (non-significant when age of onset was controlled) indicating good predictive validity for the measure in assessment of alcohol related consequences and alcohol dependence. | Test-retest reliability (fair) Predictive validity (fair) |
|  | Hilton (1989)  Short-term recall measure (10 week recall). Measure broken up into 10 weekly instalments mailed to participants.  Graduated-frequency measure (30 day recall). Contained questions asking how many days out of 30 respondent drank in amount ranges 1-2 drinks, 3-4 drinks, 5-7 drinks, 8-11 drinks, 12 or more drinks.  Beverage specific Quantity-frequency measure (2 week recall). Questions included days drinking beer, wine or liquor respectively, and they amount of ounces they estimate they consumed. | Hypothesis validity (convergent validity) | Convergent validity Correlation between 10 week short-term recall and 30 day graduated-frequency measure ranged from; volume of drinks correlation 0.89, frequency of alcohol consumed 0.68, 12 or more drinks consumed 0.63, days 8-11 drinks consumed 0.69, days 5-7 drinks consumed 0.59, days 3-4 drinks consumed 0.50, days 1-2 drinks consumed 0.61. Correlations between mean recall calculated from the 10 week short-term recall and 2 week quantity-frequency measure were positive at; 0.88 for volume of drinks consumed, 0.85 for days of beer consumed, 0.89 for days of beer usually consumed, 0.80 for days of wine consumed, 0.66 for days of wine usually consumed, 0.81 for days of liquor consumed and 0.65 for days of liquor usually consumed. Moderate to good convergent validity between measures was reported. | Convergent validity (fair) |
|  | Koppes et al (2002)^a^  Quantity-frequency measure (ranging from never to daily intake). Respondents were asked how often they consumed 1 or more alcoholic drinks. Frequency options were never, monthly or less often, two or four times per month, two or three times per week, or four times per week or more often. Respondents were then asked how many alcoholic drinks (glasses) they consumed on an average drinking day. Quantity options were 1, 2, 3, 4-5, 6-8 or 9 or more.  Short-term recall measure (dietary history interview). A trainer interviewer asked respondents what, how often and how much they ate and drank (including alcohol consumed) during each day during the past month. | Criterion validity (concurrent validity) | Concurrent validity  The recall interview was reported as a criterion standard. Correlation coefficients between the two measures were reported to be 0.77 for men and 0.87 for women. Good correlation between the quantity-frequency and dietary history interview short-term recall measures which indicates good concurrent validity. | Criterion validity (poor) |
|  | LaBrie et al (2004)  Short-term recall measure (monthly TimeLine follow back method). Respondents asked to recall alcohol consumption over the past month using a blank calendar where they are instructed to indicate the days they consumed alcohol with number of drinks on those days. | Hypothesis validity (convergent validity) | Convergent validity  The measure correlated positively on total drinks in month (0.65), average drinks per month (0.69) and drinking days per month (0.52). There were no significant differences found between measures administered individually with regards to drinking days (difference of 0.21) and drinks consumed each drinking occasion (difference of 0.17). The quantity-frequency measure and Timeline Follow back Method yielded strongly correlated values (p<0.001) with coefficients between 0.52-0.69 showing moderate convergent validity. | Convergent validity (fair) |
|  | Northcote and Livingston^a^ (2011)  Short-term recall measure (last occasion self-report of drinks consumed). Participants were asked to recall alcohol intake consumed as standard drinks from a night out. | Criterion Validity | Criterion validity  Comparing mean observed and self-reported drinks reported mostly significant associations with p values of 0.6, 0.31, 0.04 and <0.01 for: up to 4 drinks, 5-8 drinks, 9-12 drinks and more than 12 drinks respectively. The short-term recall measure showed good criterion validity for respondents consuming ≥9 drinks. . | Criterion validity (poor) |
|  | O’Hare et al (1991)  Weekly graduated-frequency measure. Respondents were asked how often they usually drank alcoholic beverages, with responses ranging from never to 7 or more times per week. 1-2 drinks, 3-4 drinks, 5-7 drinks, 8-10 drinks, 11-15 drinks or 16 or more drinks were the quantity items.  Short-term recall measure (retrospective recall of past 7 day alcohol intake). Respondents were asked to fill in boxes provided for each of the previous 7 days with the number of drinks consumed on each day. | Hypothesis validity (convergent validity) | Convergent validity Correlations between the graduated-frequency and retrospective recall measures were significant at 0.74, with gender specific correlations for men as 0.79 and women 0.60.  Moderate to good convergent validity was reported. | Convergent validity (good) |
|  | O’Hare et al (1997)^a^  Typical weekly Graduated-frequency Measure. Respondents were asked how often they drank ranging from never to seven or more times per week. Quantity items ranged from 0 drinks to 16 or greater per occasion.  Short-term recall measure (retrospective recall of past 7 day alcohol intake). Starting with the previous day respondents were asked to fill in the number of drinks they consumed over the past 7 days. | Criterion validity  Hypothesis validity (predictive validity) | Criterion validity  The association between the recall measure and MAST (which acted as the criterion standard) was significant at p<0.01 indicating good criterion validity.  Predictive validity  Using MAST cut-off score ≥3 as a criterion variable sensitivity and specificity values were 76 and 59.8 for the recall measure. Using MAST cut off score ≥2 sensitivity and specificity values were 59.7 and 70.9. Moderate to good predictive validity was found for retrospective recall. | Criterion validity (fair)  Predictive validity (fair) |
|  | Parker et al (1996)^a^  Short-term recall measure (beverage specific past 24 hour recall). Participants were asked whether they had any alcohol to drink in the last 24 hours such as wine, beer or any type of liquor  Annual graduated-frequency measure. Respondents were asked how often in the past year they had consumed each type of alcoholic beverage. Options ranged from never or less than once per month to 6 or more times per day. | Criterion validity (concurrent validity)  Inter-rater Reliability | Concurrent validity  The short-term recall measure acted as the criterion standard. Kappa statistics reported between measures ranged from 0.08 (p<0.001), 0.38 (p<0.001) and 0.81 (p<0.001) for low, medium and high consumers of alcohol, indicating good concurrent validity for high consumers of alcohol only.  Inter-rater reliability  Kappa values for both measures were 0.28 for those who consumed any alcohol type, 0.38 for beer, 0.44 for liquor and 0.37 for wine. Inter-rater reliability was poor (below 0.70). | Criterion validity (poor)  Inter-rater Reliability (fair) |
|  | Poikolainen et al (2002)  Annual quantity-frequency questionnaire. This involved 10 drinking frequency options (never, 1-2 times per year, 3-4 times per year, approximately every second month, approximately once a month, approximately twice a month, once a week, 2-3 times weekly, 4-5 times weekly, 6-7 times weekly). Quantity questions were beverage specific per occasion with highest options including 5 or more litres of beer, 1.5 litres of wine or 1 litre of more of spirits.  Daily graduated-frequency measure. Responses ranged from 15 or more drinks per day to 1-2 drinks per day, with 1 drink described as a standard serving with an alcohol content of 12g each.  Short-term recall measure (past month recall of intake). This was a daily recall of alcohol intake. | Hypothesis validity (convergent validity) | Convergent validity Coefficients between the quantity-frequency (1 represents time 1 and 2 represents time 2) and graduated-frequency measures before and after application of the short-term recall measure. Correlation coefficients were 0.95 between the short-term recall measure and quantity-frequency 1, 0.95 between the short-term recall measure and quantity-frequency 2, 0.90 between the short-term recall measure and graduated-frequency 1 and 0.93 between the short-term recall measure and graduated-frequency 2. Convergent validity was reported as good. | Convergent validity (good) |
|  | Read et al (2006)^a^  Short-term recall measure (past 90 day intake). Respondents were asked how often they had been drunk on alcohol in the past 90 days. A standard drink was defined as 1 ounces distilled spirits, 6ounces wine or 12ounces beer and participants were asked to report their drinking in these terms. | Criterion validity (concurrent validity) | Concurrent validity  90 day recall measure frequencies of alcohol consumed correlated with a developed measure of alcohol-associated negative consequences (criterion standard) with correlation values of 0.36, p<0.001 and with quantities of alcohol consumed with an r value of 0.31, p<0.001, indicating poor concurrent validity. | Criterion validity (excellent) |
|  | Rehm et al (1999)  Quantity-frequency measure of a typical drinking occasion. Respondents were asked about their usual number of drinks consumed per drinking occasion.  Annual graduated-frequency measure. Respondents were asked to recall their highest number of drinks for any occasion in the past year, with the highest quantity item 12 or more drinks.  Short-term recall measure (past week recall). Participants were asked for the number of drinks consumed on each of the seven days before the survey, beginning with the most recent day. | Hypothesis validity (convergent validity and predictive validity) | Convergent validity  Correlations with the quantity-frequency and graduated-frequency measures and weekly drinking recall were moderate at both approximately 0.40.  Predictive validity  Lowest number of discharges from hospital (measure of alcohol-related morbidity) estimated by short-term recall compared to graduated-frequency (12% less) and quantity-frequency measures. Years of life lost (measure of alcohol-related mortality) estimated by graduated-frequency measure 22% higher than short-term recall estimate. Quantity-frequency estimate of alcohol-related mortality 13% than short-term recall estimate, indicating poor predictive validity of the three measures. | Convergent validity (fair)  Predictive validity (excellent) |
|  | Searles et al (1995)  Short-term recall measure (Daily self-report of alcohol intake). Participants were asked to record through voice interactive technology how many drinks of a particular beverage were consumed in integer values.  Short-term recall measure (annual retrospective recall). Participants asked to recall 112 day alcohol intake as quantities and frequencies. | Hypothesis validity (predictive validity) | Predictive validity  Correlations between daily self-report and retrospective recall reports was reported as a mean consumption variable which correlated with alcohol intoxication level as 0.86 and with alcohol related problems level as 0.69. Predictive validity is moderate between daily self-report and retrospective recall and alcohol related problems, and good between daily self-report and retrospective recall and alcohol intoxication level. | Predictive validity (poor) |
|  | Searles et al (2000)  Short-term recall measure (Timeline Follow back over 366 days). Individuals asked to record alcohol as number of drinks per day on a day-to-day basis.  Short-term recall measure (Daily self-report of alcohol intake). Participants were asked to record through voice interactive technology how many drinks of a particular beverage were consumed in integer values. | Hypothesis validity (convergent validity) | Convergent validity  Correlations between the daily self-report measure and Timeline Follow back measure were reported as 0.60 at 180 days of administration, 0.57 at 270 days of administration and 0.57 at 366 days of administration, indicating moderate convergent validity. | Convergent validity (fair) |
|  | Tuunanen et al (2013)  Quantity-frequency measure (typical drinks consumed per occasion). Participants asked how often they drank alcohol and how many drinks per drinking occasion. Reported drink numbers were translated into grams of absolute alcohol.  Short-term recall measure (past week recall). Respondents asked how many glasses or bottles of drinks containing alcohol they consume per week. | Hypothesis validity | Hypothesis validity  The hypothesis tested was that there would be greater alcohol consumption reported using a quantity-frequency question over mean weekly intake. Mean Weekly intake gave lower values of weekly alcohol consumption. The past week recall measure reported mean alcohol consumption (g of absolute alcohol) for all, moderate, risky and heavy episodic drinkers as 115.3, 89.1, 228.1 and 217.8g, lower than the quantity-frequency measure indicating good hypothesis validity. | Hypothesis validity (fair) |
|  | Weingardt et al (1998)^a^  Graduated-frequency measure (peak monthly alcohol consumption). Respondents asked about occasion of highest alcohol consumption in the past month. Response options for drinkers were 1-2 drinks, 3-4 drinks, 5-6 drinks, 7-8 drinks and 8 or more drinks.  Graduated-frequency measure (typical weekend quantity). Respondents were asked about their alcohol intake over a typical weekend evening during the previous month. Response options remained the same.  Short-term recall measure (typical daily quantity). Respondents asked to fill in number of drinks usually consumed for each day of the week focusing on the past month. | Criterion validity (concurrent validity)  Hypothesis validity (predictive validity) | Concurrent validity  The daily quantity measure correlated moderately with Rutgers Alcohol Problems Inventory with r value 0.57 and Alcohol Dependence Scale with r value 0.54.  Predictive Validity  The daily quantity measure classified 6.2% of drinkers as chronic and 7.4% as on an increased trajectory.  Significant variation was found between the three measures in classifying respondents as chronic and increased trajectory drinkers indicating poor predictive validity. | Criterion validity (good)  Predictive validity (good) |
|  | Whitfield et al (2004)  Annual quantity-frequency measure. Participants were asked about their frequency of alcoholic drink consumption in the past year along with usual number of drinks taken.  Short-term recall measure (past week recall of alcohol intake). Respondents completed a table indicating how many glasses of beer, wine, spirits, sherry and other alcoholic drinks they had consumed in the past week. Glass volumes were quoted for particular drinks for both measure types to standardise quantities of alcohol reported. | Test-retest reliability | Test-retest reliability  Test-retest reliability was calculated as correlations between administrations (years 1980-1993). Past week recall test-retest reliabilities from surveys were 0.54 (between 1980 and 1989) 0.60 (between 1980 and 1993) 0.57 (between 1989 and 1980) 0.67 (between 1989 and 1993) 0.56 (between 1993 and 1980) and 0.70 (between 1993 and 1989). Moderate to good test-retest reliability was reported for the past week recall measure. | Test-retest reliability (fair) |

Table Legend: Table presenting the psychometric findings of included studies and their COSMIN quality ratings assigned by review authors. Results are grouped into studies/measures, properties, study results and methodological quality assessment scores.

*CAGE* Cut down, Annoyed, Guilty, Eye-opener [45], *MAST* Michigan Alcoholism Screening Test [55]

^a^in these studies the reference standard was defined by the study authors
